# Protective Effects of a synthetic glycosaminoglycan mimetic (OTR4132) in a rat immunotoxic lesion model of septohippocampal cholinergic degeneration

**DOI:** 10.1007/s10719-022-10047-x

**Published:** 2022-03-07

**Authors:** Patricia Marques Pereira, Dulce Papy-Garcia, Denis Barritault, Franck Chiappini, Rolf Jackisch, Sarah Schimchowitsch, Jean-Christophe Cassel

**Affiliations:** 1 UMR 7364 LNCA, Université de Strasbourg, 12 rue Goethe, F-67000 Strasbourg, France; 2 Glycobiology, cell growth and tissue repair research unit (Gly- CRRET), Univ Paris Est Créteil (UPEC), F-94010 Créteil, France; 3 Organ, Tissue, Regeneration, Repair and Replacement, OTR3 (SAS), 4 rue Française, F-75001 Paris, France; 4 Institute of Experimental and Clinical Pharmacology and Toxicology, Laboratory of Neuropharmacology, University of Freiburg, Hansastraße 9A, D-79104 Freiburg, Germany; 5 Laboratoire de Neurosciences Cognitives et Adapatatives, CNRS, 12 rue Goethe, F-67000 Strasbourg, France

**Keywords:** Alzheimer, Heparan sulfate mimetic, Hippocampus, Neuroprotection, Septohippocampal pathway, OTR4132

## Abstract

Using a partial hippocampal cholinergic denervation model, we assessed the effects of the RGTA^®^ named OTR4132, a synthetic heparan-mimetic biopolymer with neuroprotective/neurotrophic properties. Long-Evans male rats were injected with the cholinergic immunotoxin 192 IgG-saporin into the medial septum/diagonal band of Broca (0.37 µg); vehicle injections served as controls. Immediately after surgery, OTR4132 was injected into the lateral ventricles (0.25 µg/5 µl/rat) or intramuscularly (1.5 mg/kg). To determine whether OTR4132 reached the lesion site, some rats received intracerebroventricular (ICV) or intramuscular (I.M.) injections of fluorescent OTR4132. Rats were sacrificed at 4, 10, 20, or 60 days post-lesion (DPL). Fluorescein-labeled OTR4132 injected ICV or I.M. was found in the lesion from 4 to 20 DPL. Rats with partial hippocampal cholinergic denervation showed decreases in hippocampal acetylcholinesterase reaction products and in choline acetyltransferase-positive neurons in the medial septum. These lesions were the largest at 10 DPL and then remained stable until 60 DPL. Both hippocampal acetylcholinesterase reaction products and choline acetyltransferase-positive neurons in the medial septum effects were significantly attenuated in OTR4132-treated rats. These effects were not related to competition between OTR4132 and 192 IgG-saporin for the neurotrophin receptor P75 (p75^NTR^), as OTR4132 treatment did not alter the internalization of Cy3-labelled 192 IgG. OTR4132 was more efficient at reducing the acetylcholinesterase reaction products and choline acetyltransferase-positive neurons than a comparable heparin dose used as a comparator. Using the slice superfusion technique, we found that the lesion-induced decrease in muscarinic autoreceptor sensitivity was abolished by intramuscular OTR4132. After partial cholinergic damage, OTR4132 was able to concentrate at the brain lesion site possibly due to the disruption of the blood-brain barrier and to exert structural and functional effects that hold promises for neuroprotection/neurotrophism.

## Introduction

After injury to the adult mammalian central nervous system (CNS), spontaneous repair is generally limited [1–4]. Nevertheless, both endogenous and exogenous neuroprotective/neurotrophic substances may prevent neurons from degeneration and/or enable regeneration [5–7]. Experimentally, *in vivo* CNS repair *via* direct or indirect effects on neuron survival and/or axon regrowth can be induced by acute, subchronic, or chronic injections of growth factors including nerve growth factor (NGF), brain-derived neurotrophic factor (BDNF), or fibroblast growth factors (FGF) [8–11]. Heparin binding growth factors (HBGF), such as FGF and transforming growth factors (TGF), protected against ischemic injuries, reducing infarct size in an animal model of stroke [10, 12–17]. However, administration of exogenous growth factors may induce adverse effects due to penetration of the factors into unlesioned brain regions leading to pleiotropic effects and to immune responses [6, 18]. On the other hand, endogenously synthetized neurotrophic factors can also be released in response to injury, but generally only within or near the injured or denervated regions [14]. However, injury-related phenomena, such as enzymatic degradation, which occurs rapidly [19, 20], may limit the effects of the endogenous factors. Therefore, compounds that slow down the degradation of endogenously released neurotrophic factors, or that protect neurons from degeneration, may hold therapeutic potential.

Here, we used the glycosaminoglycan mimetic OTR4132, a member of the RGTA® family of polymers, which, by mimicking glycosaminoglycans in the extracellular matrix, protect endogenous growth factors from proteolytic degradation. Some of these polymers are currently used in the clinic as regenerative agents [9, 15, 21, 22]. By protecting HBGF from proteolytic degradation, RGTA® fostered substantial tissue repair in various *in vivo* injury models [9, 21]. Recently, we showed that OTR4132, also known as Hsm4131, administered I.V. after stroke induction in a rat model, conferred long-lasting neuroprotection and significantly reduced functional deficits [23]. It also restored the extracellular matrix and increased brain plasticity processes (i.e. angiogenesis and neurogenesis) in the affected brain hemisphere. In the same set of experiments, we additionally showed that OTR4132 was safe, and that it could specifically locate in the injured hemisphere. Moreover, the therapeutic interest of RGTA®-based therapies was shown in mice models of Alzheimer’s disease-related tauopathy in which a RGTA® prototype of small size (called F6) was administrated and blocked neuronal uptake, seeded aggregation and transcellular propagation of stereotactically injected tau fibrils [24]. These data suggest that RGTA® might offer benefits in neurodegenerative diseases where it can protect neurons from degeneration. However, the protective effect of RGTA® has not been fully assessed in neural cells, such as the cholinergic cells involved in neurodegeneration. Here, we studied the neurotrophic and neuroprotective effects of OTR4132 in a model that met the following two criteria: (i) partial cholinergic damage reproducible in terms of both severity and location, and (ii) limited post-injury sprouting of the cholinergic neurons or other spontaneous restorative mechanisms. A partial immunotoxic lesion was induced by local administration of the immunotoxin 192 IgG-saporin in the forebrain cholinergic neurons located in the medial septum (MS) and vertical limb of the diagonal band of Broca (vDBB). 192 IgG-saporin carries the ribosome-inactivating protein (saporin) combined with 192 IgG (a monoclonal antibody against rat p75^NTR^ receptors) [25, 26]. Since such receptors are specific to cholinergic neurons in the basal forebrain, 192 IgG-saporin injected intraseptally selectively damages this population of neurons [3, 27, 28]. Interestingly, recovery of choline acetyltransferase (ChAT) and acetylcholinesterase (AChE) activities after the induction of partial lesions is extremely limited in the hippocampus, even after 1 year [29–31]. Assessment of the RGTA® named OTR4132 neuroprotective effects in the 192 IgG-saporin model was conducted with several complementary experiments: evaluation of the morphological and histochemical effects of OTR4132 administered directly into the cerebral ventricles or by intramuscular (I.M). injection, followed by the assessment of the neuroprotection effects of OTR4132 after induction of larger lesions. Next, the effects of OTR4132, which is a heparan sulfates/heparin mimetic, were compared to those of heparin. Then, competition between OTR4132 and 192 IgG for the p75NTR receptor was investigated. Finally, additional information about the effects of OTR4132 on the function of cholinergic terminals arising from a partially damaged septum, were obtained using a hippocampal-slice superfusion model. The effect of OTR4132 on the muscarinic modulation of electrically evoked acetylcholine (ACh) release [32, 33] was determined using different pharmacological approaches: inhibition of the acetylcholinesterase (with Physostigmine) associated or not to a muscarinic antagonist (Atropine) or a muscarinic agonist (Oxotremorine), which inhibit the ACh overflow, and a selective serotonin receptor agonist, CP-93,129, selective for the 5-HT_1B_ receptors. Indeed, 5-HT_1B_ receptors are widely distributed in striatum and hippocampus, and oppositely regulate the excitability of cholinergic interneurons by inhibition of ACh release from cholinergic neurons [34, 35].

## Materials and methods

### Animal care

Animal care and procedures were conducted in conformity with our institution’s guidelines. 3-month-old Long-Evans male rats were obtained from R. Janvier (C.E.R.J., Le Genest-St-Isle, France). They were housed individually in transparent Makrolon cages (42 × 26 × 15 cm) placed in rooms on a 12 h-12 h light-dark cycle (lights on at 7:00 am) under controlled temperature (21 °C). Food and water were available *ad libitum*. All procedures have been performed in compliance with 3R’s ethical rules on animal experimentation and in accordance with the European Directive concerning animal experimentation (2010/63/EU). The protocol was submitted to the Ministry of Higher Education and Research to obtain the ethic approval for using animals for scientific purposes (Animal protocol numbers: 67-215 and 67-101 for J-CC and SS, respectively under their authorities). Every effort was made to minimize suffering and the number of animals was as small as possible, ranging from 3 to 5 given the experimental and statistical constraints.

### Induction of the hippocampal lesion

Procedures, dosages, and time intervals required to detect morphological effects of the immunotoxin were determined in preliminary experiments using the same batch of 192 IgG-saporin (batch 21#107; ATS, San Diego, CA) [2]. This batch was used in all experiments. The size of the immunolesion was monitored using AChE histochemistry in the hippocampus and anti-ChAT immunohistochemistry in the septal area (see below). All surgical procedures were performed under aseptic conditions. A mixture of ketamine (98 mg/kg intraperitoneally) and xylazine (13 mg/kg intraperitoneally) was used for anesthesia. A stereotactic method was used to inject 192 IgG-saporin into the MS. The 192 IgG-saporin dose was 0.37 µg for the induction of a partial lesion (0.46 µg/µl, 0.2 µl/site, 0.1 µl/min, in sterile artificial cerebrospinal fluid) and 0.60 µg for the induction of an extensive lesion (0.75 µg/µl, 0.2 µl/site, 0.1 µl/min). Control animals were injected with artificial cerebrospinal fluid (aCSF, Harvard Apparatus). After the fur was shaved, the scalp was prepared using povidone-iodine and alcohol 70%, and the rats were placed into a stereotaxic device (Kopf Instruments) with lambda and bregma at the same vertical coordinate. A small midline incision was made over the scalp to provide access to the cranium, and the cranial surface was cleaned with oxygenated water. Coordinates for cannula placement to target the MS were anteroposterior to bregma 0.6 mm (AP= + 0.6) and 0.20 mm lateral to the midline (L= ± 0.2). A skull window was outlined with a fine drill, and a stainless steel cannula was lowered until it reached the meninx to establish “0”. The cannula was subsequently placed at vertical (i.e. dorso-ventral coordinate) – 7.2 mm and – 6.5 mm (V= – 7.2, and – 6.5) [36]. The injections were given through a stainless steel cannula, which was left *in situ* for 5 min after each injection to enable diffusion of the toxin. The cannula was then slowly removed. The scalp was sutured with sterile Vicryl*Plus 2-0 (Ethicon, Johnson & Johnson). After surgery, each animal was allowed to recover from anesthesia under a warm lamp before returning to its cage and the animal facility.

Brains for each following treatments were harvested at 4, 10, 20 or 60 days post-lesion (DPL).

### Drug treatments

**Fig. 1:**
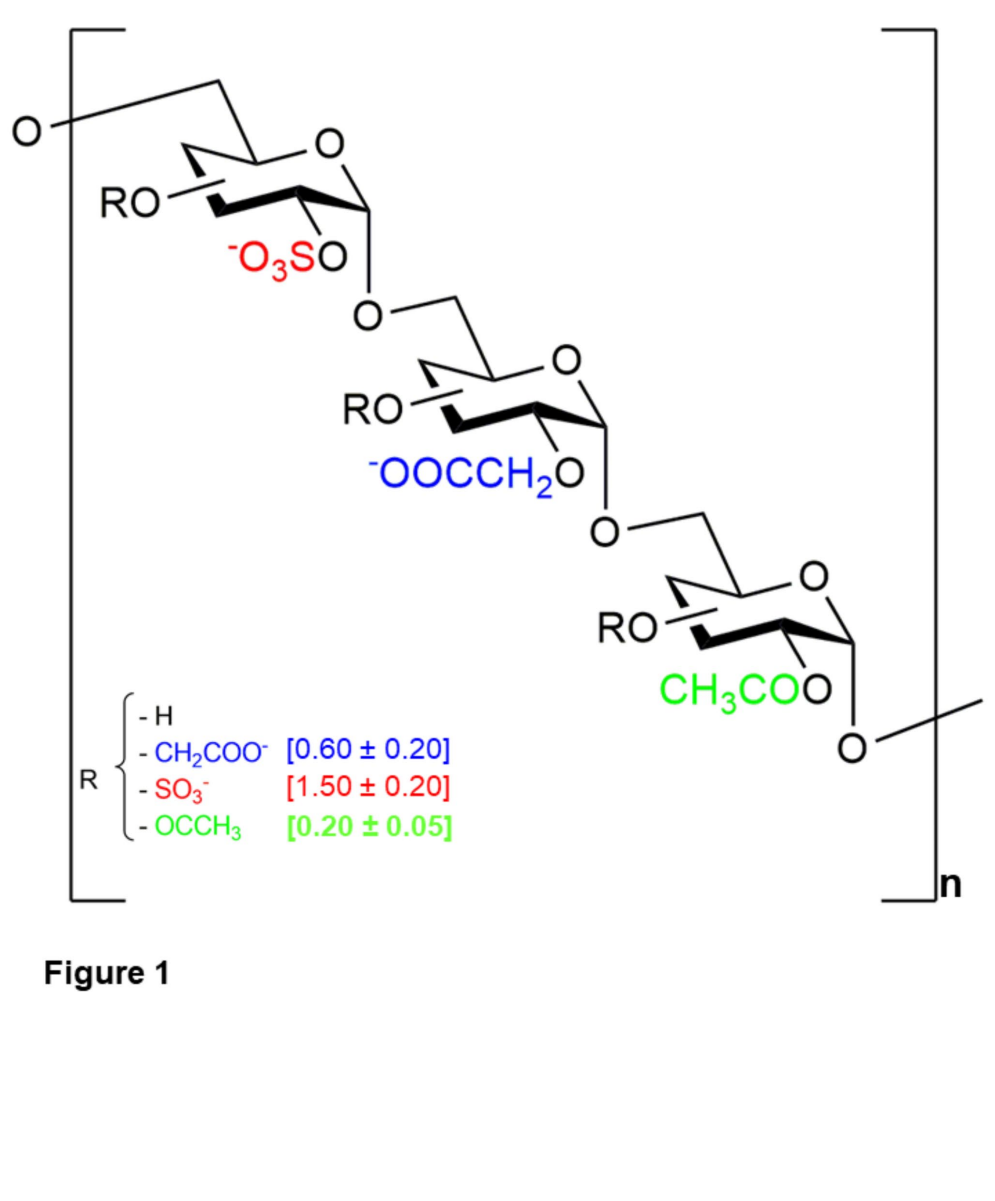
**Chemical structure of OTR4132 molecule.** OTR4132, also known as Hsm4131 [23], is a sodium salt of α-1,6 poly(carboxymethylglucose acetyl sulfate) corresponding to the following molecular structure [((C_6_H_10_O_3_)_n_)-(O*-*(CH_2_-CO_2_^−^)_0.6n_)-(O-(-SO_3_^−^)_1.4n_)-(O-(CH_3_CO^−^)_0.2n_), Na^+^] and with a molecular weight (MW) ranging between 80 and 130 kDa (i.e. Theorical cacluclated: MW_mean_= 87 kDa ± 8 kDa). OTR4132 is a dextran 40 derivative (α1-6 glucopyranose polymer), statistically substituted by carboxymethyl (-CH_2_-COO^−^), sulphate (-SO_3_^−^) and acetyl (-OCCH_3_) groups with a degree of substitution of 0.60 ± 0.20, 1.50 ± 0.20 and 0.20 ± 0.05, respectively, and represented into the brackets (CAS RN: 2342614-00-8). R is the proportion of each substituted group at both carbons C-3 and the C-4 positions. Each substituted group is randomly distributed on the glucopyranose units [37].

The heparan sulfate mimetic RGTA® named OTR4132 (Batch# 027816109P539, OTR3 SAS, Paris, France) is a synthetic derivative of dextran 40, composed of about 240 glucosidic units linked by α1-6 bonds. OTR4132 molecule (CAS RN: 2342614-00-8), polymeric structure also named Hsm4131 [23], is characterized by the average degrees of substitution (ds) of carboxymethyl (dsCM= 0.60 ± 0.20), sulfate (dsS= 1.50 ± 0.20) and acetate (dsAc= 0.20 ± 0.05) groups carried by glycosidic units (Fig. 1), as confirmed by acidimetric titration, elementary analysis, and ^1^ H NMR spectrometry, as described in previous work [23, 37].

Fluorescein-labeled OTR4132 was prepared by incubating a solution of OTR4132 (250 mg, 6.25 µmol, in 1 ml of aqueous buffer, pH= 7.5) with fluorescein-5- thiosemicarbazide (FTSC, 53 mg, 125 µmol, from Molecular Probes/Invitrogen) at 30 °C for 24 h. Under these conditions, only the single reducing glucose unit present in OTR4132 can be labeled without affecting the global structure of the drug [37–40]. After purification by ultrafiltration, the FTSC-derived-OTR4132 was obtained as a powder and conserved under dry conditions as previously described [37].

### OTR4132 treatment

OTR4132 was injected immediately after the induction of hippocampal lesion by a single intracerebroventricular (ICV) injection or by a single I.M. injection as follows.

#### ICV OTR4132 treatment

OTR4132 was injected within the lateral cerebral ventricles (50 µg/ml, 5 µl/site, 0.5 µl/min). Coordinates for ICV injection, in mm from bregma, were AP= - 0.8, L= ± 1.5, and V= - 4.3 [36].

#### I.M. OTR4132 treatment

To explore the neuroprotector/neurotrophic effects of OTR4132 on 192 IgG-saporin specific cholinergic hippocampal lesions, sterile saline OTR4132 solution was injected I.M. at 1.5 mg/kg based on previous data [23, 41–44].

In other experiments, FTSC-labeled OTR4132 was injected I.M. (1.5 mg/kg) as well, to determine whether OTR4132 crossed the blood-brain barrier (BBB), penetrated, and remained within the lesion site.

Finally, to explore a possible neuroprotective/neurotrophic effect of I.M. injected OTR4132 in animals with larger lesions, rats were given a higher dose (0.60 µg/rat) of 192 IgG-saporin immediately followed by I.M. OTR4132 injection (1.5 mg/kg).

### Other Treatments

For comparison, heparin (0.8 IU/ml ICV or 16 IU/ml I.M.) was injected using the same protocol as for OTR4132 treaments.

For competition between OTR4132 and the antibody 192 IgG for the p75-NTR receptor, a mixture of 5 µl OTR4132 (same dose as for ICV administration, i.e. 0.25 µg/site) and 2 µl of 192 IgG-Cy3 (0.37 µg/site) were injected intraseptally according to the above-described procedure for lesion induction.

### Long-term OTR4132-mediated recovery

The effects of 192 IgG-saporin on septo-hippocampal cholinergic neurons and the potential OTR4132-mediated recovery were evaluated 1 month after lesion induction using neuropharmacological measurements of various cholinergic functions in the hippocampal structure as described hereinafter. One-quarter of the ventral left and right hippocampi parts were pooled and homogenated for measurement of cholinergic markers such as acethylcholinesterase and choline acetyltransferase activities. The remaining parts of hippocampi were used to prepare slices for *in vitro* evaluation of electrically evoked ACh-release in superfusion experiments. Controls for each experiment received sterile sodium salt solution (0.9% NaCl) instead of OTR4132.

### Pharmacological approaches for testing ACh release: Tissue preparation

For all histological, histochemical, and immunohistochemical experiments, rats were given an overdose of sodium pentobarbital (100 mg/kg) and perfused transcardially with 60 ml of 4% phosphate-buffered formaldehyde (pH= 7.4; 4 °C) at the different DPL. The brains were then dissected, post-fixed in the same fixative for about 2 h, washed with 0.1 M PBS, and transferred into a phosphate-buffered 20% sucrose solution for 36-40 h at 4 °C. The brains were then quickly frozen in isopentane and subsequently cut in a cryostat.

### Immunochemistry of ACh ans ChAT expressions

#### Tissue preparation

Coronal Sect. 30 μm in thickness were collected onto gelatin-coated slides. For rats treated with FTSC-labeled OTR4132, sections were mounted with anti-fading medium and stored at 4 °C in the dark until examination. For other experiments, sections were dried at room temperature for 36 h and stained for AChE according to Koelle’s revised protocol [45, 46]. Ethopropazine (0.3 mM; Sigma, St-Louis, MO) was used to block non-specific cholinesterases, and acetylthiocholine iodide (4 mM; Sigma, St. Louis, MO) was used as the substrate. The details of the following procedures have been described in our previous works [47, 48].

#### Evaluation of acetylcholinesterase staining

The extent of cholinergic innervation, proportional to the amount of AChE-positive reaction products in the hippocampus [49, 50], was determined using a computer-assisted image analysis system (SAMBA Technologies, Paris, France) coupled to a monochrome CCD digital video camera (Model XC 77CE, Sony, Shiga, Japan) equipped with a 60-mm Nikkor objective and a Triplux extension tube (Nikon, Paris, France). Optical density (O.D.) was measured on digitized images by bilaterally delineating the hippocampal region on two sections at the same level of anteriority (AP=-3.14 and AP=-5.30 from bregma, respectively). For digitization, sections were placed on a Kaiser Prolite 5000 light box (Kaiser Fototechnik, Buchen, Germany). Magnification from section to computer screen was 2.5. The mean O.D. of the central part of the corpus callosum within the same brain section was taken as the background level and was subtracted from all measures before analysis. The investigator performing the O.D. measurements was blinded to the group assignment.

#### Choline acetyltransferase immunostaining in the septal region

Cryostat sections at 60 μm thickness were collected and kept free floating at -20 °C in cryopreservative medium until immunolabeling was performed. The sections were washed several times in 1 M PBS, pH= 7.4, then soaked for 1 h in 10% normal donkey serum (Dutscher, Brumath, France) in PBS containing 0.5% Triton X-100 and 0.2% merthiolate. Sections were incubated overnight at room temperature with a goat polyclonal antibody against ChAT (1:200, Chemicon International, Temecula, CA), then incubated for 1 h with biotinylated secondary antibody with peroxidase against goat IgG (1:200; Chemicon International, Temecula, CA). Sections were soaked for 1 h in ABC Vectastain Elite kit (1:500; Vector Labs, Burlingame, CA) before using a diaminobenzidine kit (Vector Labs, Burlingame, CA) to reveal positive neurons. Brain sections were then mounted onto gelatin-coated slides, dried, dehydrated, and cover-slipped. Omission of the primary antibody served as the negative control and resulted in no detectable staining.

#### Determination of choline acetyltransferase-positive neuron counts

At the appropriate level of anteriority, the anterior commissure was used as an anatomical landmark to select, define, and standardize the location of counting frames of a predefined size in the MS. This frame was defined ventrally by an imaginary line through the center of the anterior commissure, and both dorsally and laterally by the distribution of ChAT-positive neurons, as described in detail elsewhere [51]. Three coronal sections per animal were selected: one section corresponding to the injection site (AP + 0.6 mm, as determined by the cannula track) and, in order to assess immunotoxin diffusion, two sections located within 0.4 mm from the injection site.

### Determination of choline acetyltransferase and acetylcholinesterase activities

Rats used for superfusion experiments were sacrificed by decapitation using a custom-made guillotine, after carbon dioxide inhalation, one month after lesion induction. Quick removal of the brain was followed by the dissection of the hippocampus from each hemisphere. The dorsal three-quarters of the hippocampus was used to prepare slices for release experiments (see below). The remaining ventral piece was homogenized in 0.5 ml of 0.32 M sucrose (in 2.5 mM HEPES, pH= 7.4) using a Potter Elvehjem glass/Teflon homogenizer (eight strokes at 500 rpm). From this crude homogenate, a 40-µl sample was taken, diluted with 360 µl of 0.1 N NaOH and used for protein assay. A 100-µl sample of the homogenate was stored at -80° C until determination of ChAT and AChE activity as previously described [47, 48].

### Assessment of acetylcholine release

#### Accumulation of [^*3*^ H]acetylcholine overflow

Accumulation and electrically-evoked release of [^3^ H]-acetylcholine overflow was assessed as previously described [47, 48]. Briefly, coronal slices (300 μm thick) of the dorsal hippocampus were prepared using a McIlwain tissue chopper (Model TC752, Mickle Laboratory Engineering Co. Ltd., UK). The slices were distributed into Petri dishes containing 2 ml modified Krebs–Henseleit (KH) buffer (Sigma-Aldrich, L’lsle D’Abeau, France) supplemented with [^3^ H]-choline (0.1 µM). The KH buffer had the following composition NaCl 118 mM, KCl 4.8 mM, CaCl_2_ 1.3 mM, MgSO_4_ 1.2 mM, NaHCO_3_ 25 mM, KH_2_PO_4_ 1.2 mM, glucose 10 mM, ascorbic acid 0.6 mM, and Na_2_EDTA 0.03 mM, pH= 7.4. The dishes were incubated under gentle agitation for 45 min at 37 °C in an atmosphere of 5% CO_2_ in O_2_ (Carbogen, Riom, France). Following incubation, the slices were transferred into superfusion chambers (12 chambers per superfusion apparatus, 1 slice per chamber) and superfused with oxygenated KH buffer (37 °C) at a rate of 1.2 ml/min. The superfusion medium was supplemented routinely with hemicholinium-3 (10 µM) to block the re-uptake of [³H]choline resulting from the degradation of released [³H]-ACh. A first electrical field stimulation (18 rectangular pulses at 3 Hz, 2 ms, 4 V/chamber, 26–28 mA) was applied 15 min after the start of the superfusion. Fractions (2 min, 2.4 ml) were collected from 32 min of superfusion onward. [^3^ H]-ACh release was induced by up to three electrical-field stimulations (90 rectangular pulses at 3 Hz, 2 ms, 4 V/chamber, 26–28 mA), after 34 min (S_1_), 50 min (S_2_), and 66 min (S_3_) of superfusion. Drugs to be tested were added to the superfusion medium of some chambers from 8 min before S_2_ and S_3_ onward, in increasing concentrations (indicated on the abscisses in the corresponding figures) from S_2_ to S_3_. Control slices were superfused throughout with drug-free KH buffer. At the end of the experiment (after 74 min of superfusion), radioactivity in the superfusate samples and slices (dissolved in 250 µl Solvable®, Perkin-Elmer, Rodgau, Germany) was determined by liquid scintillation counting. Accumulation of [^3^ H]-choline was defined as the tissue tritium content determined immediately before the beginning of fraction collection. Since hippocampal slices were very similar in size and thickness, these values provided an indirect indication of the density of the corresponding cholinergic axon terminals in the hippocampus. The fractional rate of tritium outflow was calculated as (pmoles tritium outflow per min) × 100 / 2 × (pmoles tritium in the slice at the start of the corresponding 2-min period). The stimulation-evoked overflow of tritium ([^3^ H]-overflow) was calculated by subtraction of the basal outflow and was expressed either in absolute terms (‘nCi’) or in relative terms (percent of the tritium content of the slice at the onset of the relevant stimulation period). Effects of drugs added before S_2_ (or S_3_ when indicated) were determined as the ratio of the overflow evoked by the corresponding stimulation period (S_n_/S_1_) and normalized according to the appropriate control ratio (100%, no drug addition before the corresponding stimulation). Values significantly greater than 100% indicated facilitation and values significantly smaller than 100% indicated inhibition of evoked ACh release.

### Pharmacological treatements and superfusion conditions of brain slices

Release of ACh was measured by a [^3^ H]-ACh superfusion, as previously described [52]. Briefly, [^3^ H]-ACh release was induced by three field stimulations (360 pulses, 3 Hz, 4 V per chamber, 24 mA, 2ms) applied 34 mn (S1), 50 mn (S2) and 66 mn (S3) in brain slices (300 μm thick) from control rats or rats treated with 192 IgG-saporin, receiving OTR4132 (I.M. injections in NaCl 0.9%) or control (NaCl 0.9%). Thus, 8 min before the second (S2) and third (S3) stimulation, the superfusion buffer was replaced by a buffered solution containing the drug at the chosen concentrations: (1) An indirect muscarinic inhibitor as well as an AChE inhibitor, physostigmine (Physostigmine salicylate, EP, P1600000, Sigma-Aldrish) was used at 1 µM (S2) alone or co-applied with atropine (Atropine sulfate, EP, A1400000 Sigma-Aldrish), a muscarinic antagonist, at 1 µM (S3). (2) A muscarinic agonist, Oxotremorine (Oxotremorine methiodide, Sigma-Aldrish) was used at four concentrations: 0.01, 0.1, 1 and 10 µM. (3) A selective 5-HT_1B_ receptor agonist, CP-93,129 (1,4-dihydro-3-(1,2,3,6-tetrahydro-4-pyridinyl)-5 H-pyrrolo[3,2-b]pyridin-5-one dihydrochloride, Sigma-Aldrich) was used at four concentrations: 0.01, 0.1, 1 and 10 µM [53]. Superfusion was stopped 8 min after the last stimulation (74 min after the start of the experiment). Each slice was removed from the superfusion chamber and dissolved in 2 ml of Toluene (Packard, Frankfurt, Germany). Then, 4 ml of scintillation liquid (Ultima Gold™ LSC cocktail, Packard, Frankfurt, Germany) was added to this mixture and the radioactivity was counted in a counter (Packard Model 5003 Cobra II Auto Gamma Counter, Packard, Frankfurt, Germany). Detail of this procedure has been described [47, 48].

### Statistical analyses

O.D. data were first analyzed using one-way analysis of variance with side (left or right) as the factor. O.D. and ChAT data from 4 DPL were analyzed using two-way ANOVA with Group (Sham or Lesioned) and Drug (OTR4132 or NaCl) as the factors. This analysis was performed separately from the analyses of the other three DPL, because at 4 DPL the damaging effects of 192 IgG-saporin were not yet at their maximum level [54]. O.D. and ChAT data from 10, 20, and 60 DPL were analyzed using three-way ANOVA with Group, Drug and DPL as the factors. As the variances of some measures did not satisfy the criterion of homogeneity, square root transformation of the data was performed prior to running the ANOVA, as described elsewhere [55], and post-hoc Tukey test performed. Type I error was set-up at 5% (i.e. α = 0.05). Data are represented as mean ± standard deviation (SD).

## Results

### OTR4132 protects cholinergic neurons from partial lesion of hippocampus at the septal anterior and posterior levels, and cholinergic neurons from the medial septum

**Fig. 2:**
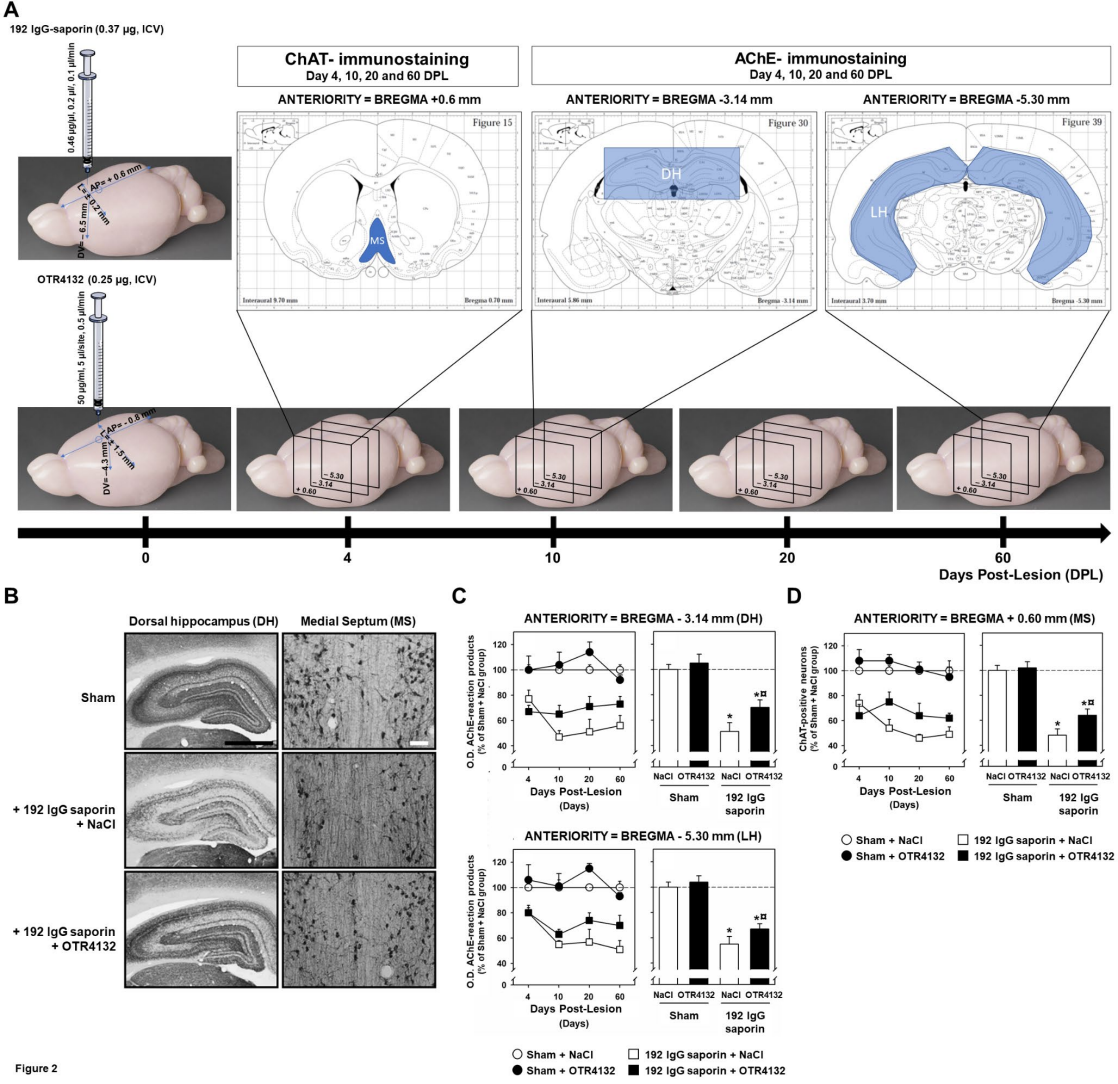
**Effects of ICV OTR4132 administration after partial lesion of septal cholinergic neurons.** (**A**) Illustration of the experimental design. Rats were first either injected with aCSF or 192 IgG saporin (0.37 µg, ICV), and then injected with OTR4132 (0.25 µg, ICV). Brains were harvested 4, 10, 20 and 60 days post-lesion (DPL) and ChAT- and AChE-immunostainings were performed at the medial septum (+ 0.6 mm) and hippocampus sections of each brain (-3.14 mm and -5.30 mm) according to the brain rat stereotaxic coordinates [36]. (**B**) Representative photomicrographs of cryostat sections through the dorsal hippocampus (left panels) or medial septum (right panels) from rats subjected to a sham operation (top panels), or after intra-septal injection with 192 IgG-saporin inducing immunolesion followed immediately by intracerebroventricular (ICV) NaCl 0.9% injection (middle panels), or by ICV OTR4132 injection (bottom panels). Black scale bar= 2.0 mm; white scale bar= 50 μm. (**C**) Quantification of photomicrographs from (**B**, dorsal hippocampus) by optical density (O.D.) assessment of acetylcholinesterase (AChE)-positive neurons in coronal brain sections through the dorsal hippocampus at an anterior (AP= -3.14 mm; top panels) or posterior (AP= -5.30 mm; bottom panels) levels in the four groups of rats including rats with sham surgery injected with NaCl or OTR4132, or rats with 192 IgG-saporin lesion followed immediately by ICV injection of NaCl 0.9% solution or OTR4132 solution. Top panels represent the percentages of the mean O.D. compared to Sham + NaCl group of the data collected at days 4, 10, 20, and 60 post-lesion. Right panels represent the data from left panels were collapsed for each group at either the anterior (AP= -3.14 mm) or the posterior (AP= -5.30 mm) level, respectively. (**D**) Quantification of photomicrographs from (**B**, medial spetum) by O.D. assessment of ChAT-positive neurons present in the medial septum (AP= +0.6 mm) in the same groups of rats as in (**C**). Left panel represents the data collected at day 4, 10, 20 and 60 post-ICV injection [90]. Right panel represents the data from A1 that have been collapsed for each group. Data are represented as mean ± SD. of the percentage (%) of O.D compared to the sham group injected with NaCl. The number of rats per group is four (n= 4). * Indicates a significant effect of the lesion compared to Sham groups injected with NaCl, *p-value*< 0.05, ANOVA test. ¤ Indicates a significant on effect of OTR4132 ICV administration in rats with 192 IgG saporin compared to 192 IgG saporin group of rats treated with NaCl. *p-value*< 0.05. ANOVA-test with post-hoc Tukey test. AChE: acetylcholinesterase; ChAT: choline acetyltransferase; DH: dorsal hippocampus; LH: lateral part of the hippocampus; MS: medial septum.

In order to detect the partial 192 IgG-saporin lesions and recovery after OTR4132 ICV administration (0.25 µg/5 µl/rat in lateral ventricle), hippocampal slices were stained for AChE-positive fibers, and medial septal slices were immunostained for ChAT-positive neurons (Fig. 2A). In the dorsal hippocampus (Fig. 2B**)**, AChE staining was markedly decreased in the dentate gyrus and CA1 layer of the lesioned rats compared to the sham-operated rats (Fig. 2B, Sham and 192 IgG-saporin lesion, left panel). The decrease was less pronounced in lesioned rats treated with OTR4132 (Figs. 2B and 192 IgG-saporin lesion + OTR4132, left panel). Similarly, the number of ChAT-positive neurons in the MS (Fig. 2B, right panel**)** was reduced after lesion induction by 192 IgG-saporin injection (Fig. 2B, middle right panel) compared to sham-operated rats receiving NaCl solution (Fig. 2B, top right panel). Reduction after OTR4132 treatment was smaller (Fig. 2B) compared to 192 IgG-saporin injected rat (Fig. 2B, bottom righ panel ). These results were confirmed by measurement of AChE staining in hippocampal slices (Fig. 2C) and by the determination of ChAT-positive neuron counts per section (Fig. 2D). The O.D. of AChE reaction products was measured in the right and left hippocampus at an anterior level (Fig. 2C; AP= -3.14 mm from bregma, top panel) and a posterior level (Fig. 2C; AP= -5.30 mm from bregma, bottom panel). Moreover, we looked for an effect of side (right or left) that might produce asymmetry of the immunotoxic damage. Side had no significant effect. Therefore, the mean of the values on the two sides was used in subsequent analyses. Interestingly, after 4 DPL, ANOVA revealed a significant Group effect (Sham, Lesioned: F 1/20= 19.6, *p-value*< 0.01), but no significant Drug effect (NaCl, OTR4132: F 1/20< 1.0) and no significant interaction between the two factors (F 1/20< 1.0), as shown in Fig. 2C on the top right panel. The Group effect reflected significantly lower AChE O.D. values in the lesioned animals compared to their sham-operated counterparts (approx. -30%). At the three other times after lesion, ANOVA showed a significant Group effect (F 1/59= 122.5, *p-value*< 0.001), a significant Drug effect (F 1/59= 8.1, *p-value*< 0.05), and a significant Group-Drug interaction (F 1/59= 3.9, *p-value*= 0.05). There was no significant DPL effect (F 2/59< 1.0), and none of the other interactions was significant. Then, the Group-by-Drug interaction was ascribable to significantly higher AChE O.D. values in OTR4132-treated lesioned rats compared to their saline-treated counterparts (ANOVA test; *p-value*< 0.001), whereas the difference between OTR4132 and saline treatment in Sham rats was not significant.

These results indicate that OTR4132 ICV administrated (0.25 µg/5 µl/rat in lateral ventricle) protects hippocampus cholinergic neurons at the septal anterior level.

Since OTR4132 significantly protect cholinergic neurons at the septal posterior level from partial hippocampus lesions, we investigated if the OTR4132 protective effect could also be observed at the septal posterior level (Fig. 2C, bottom panels). The results were very similar to those obtained at the anterior level (Fig. 2C, top panels). Indeed, at 4 DPL, two-way ANOVA showed a significant Group effect (F 1/20= 9.5, *p-value*< 0.01, ANOVA-test) but no significant Drug effect (F 1/20< 1.0), and no significant interaction between the two factors (F 1/20 <1.0). At the other DPLs, significant Group (F 1/59= 127.6, *p-value*< 0.001) and Drug (F 1/59= 6.8, *p-value*< 0.05, ANOVA-test) effects were found, as well as a significant Group-Drug interaction (F 1/59= 4.2, *p-value*< 0.05, ANOVA-test), with no significant overall DPL effect (F 2/59= 1.4). Multiple comparisons showed that these effects (Fig. 2C, bottom panels) were ascribable to the same differences as for the anterior level (Fig. 2C, top panels).

These results indicated that OTR4132 ICV administrated (0.25 µg/5 µl/rat in lateral ventricle) significantly protected hippocampus cholinergic neurons at the septal posterior level.

Because the majority of cholinergic projections target the hippocampus and the entorhinal cortex in the basal forebrain complex, specifically the medial septum (MS),In order to evaluate 192 IgG-saporin diffusion and the effect of OTR4132 ICV injection (0.25 µg/5 µl/rat in lateral ventricle) on these cholinergic neurons, ChAT-immunostaining in the MS and vertical limb of the diagonal band of broca was performed, and the number of ChAT-positive neurons was measured at the injection site (AP= + 0.6 mm; Fig. 2D). The data showed that OTR4132 significantly protects cholinergic neurons from partial lesion of hippocampus at the medial septum and vertical limb of the diagonal band of Broca (Fig. 2D, right panel).

Four (4) days after injection (4 DPL), ChAT-immunostaining showed a significant Group effect with a lower number of ChAT-positive neurons in the lesioned rats compared to the sham-operated rats (approx. -30%; F 1/19= 19.2, *p-value*< 0.001, ANOVA-test) with no significant Drug effect (F 1/19< 1.0) and no significant interaction between the two factors (F 1/19= 1.2). At later DPLs, the results showed a significant Group effect (approx. - 41%; F 1/59= 144.2, *p-value*< 0.001, ANOVA-test), a significant Drug effect (F 1/59= 8.6, *p-value*< 0.05, ANOVA-test) and a significant Group-Drug interaction (F 1/59= 7.1, *p-value*< 0.05, ANOVA-test). There was no significant overall DPL effect (F 2/59= 1.98) and none of the other interactions was significant. The Group-by-Drug interaction was ascribable to significantly higher counts of ChAT-positive neurons in OTR4132-treated lesioned rats compared to their saline-treated counterparts; the difference was not significant in the sham-operated rats. Data from 10, 20, and 60 DPL are illustrated in Fig. 2D. At AP= +1 mm and AP= + 0.2 mm from bregma, ChAT-immunostaining showed a significant Group, Drug, and DPL (10, 20, or 60 days) effect (F1/59= 62.8; F1/59= 69.8; *p-value*< 0.001, three-way ANOVA-test) and a significant Drug effect (F1/59= 8.9; *p-value*< 0.05, ANOVA-test). However, at 0.2 mm from bregma, no significant DPL effect (F2/59< 1.0) and no significant interaction among the three factors, at either level, was observed (F 2/59 <1.0 and F 2/59= 1.77) (data not shown). Since the results obtained near the 192 IgG-saporin injection site (i.e. AP= +0.6 mm) are similar, the same interpretation can be drawn here.

Taken together, the data showed that partial lesions of cholinergic neurons at the medial septum projecting to the hippocampus, induced by 192 IgG-saporin injection are significantly protected by ICV injection of OTR4132 (0.25 µg/5 µl/rat in lateral ventricle).

### OTR4132 I.M. administration protects cholinergic neurons from partial septal lesions

**Fig. 3:**
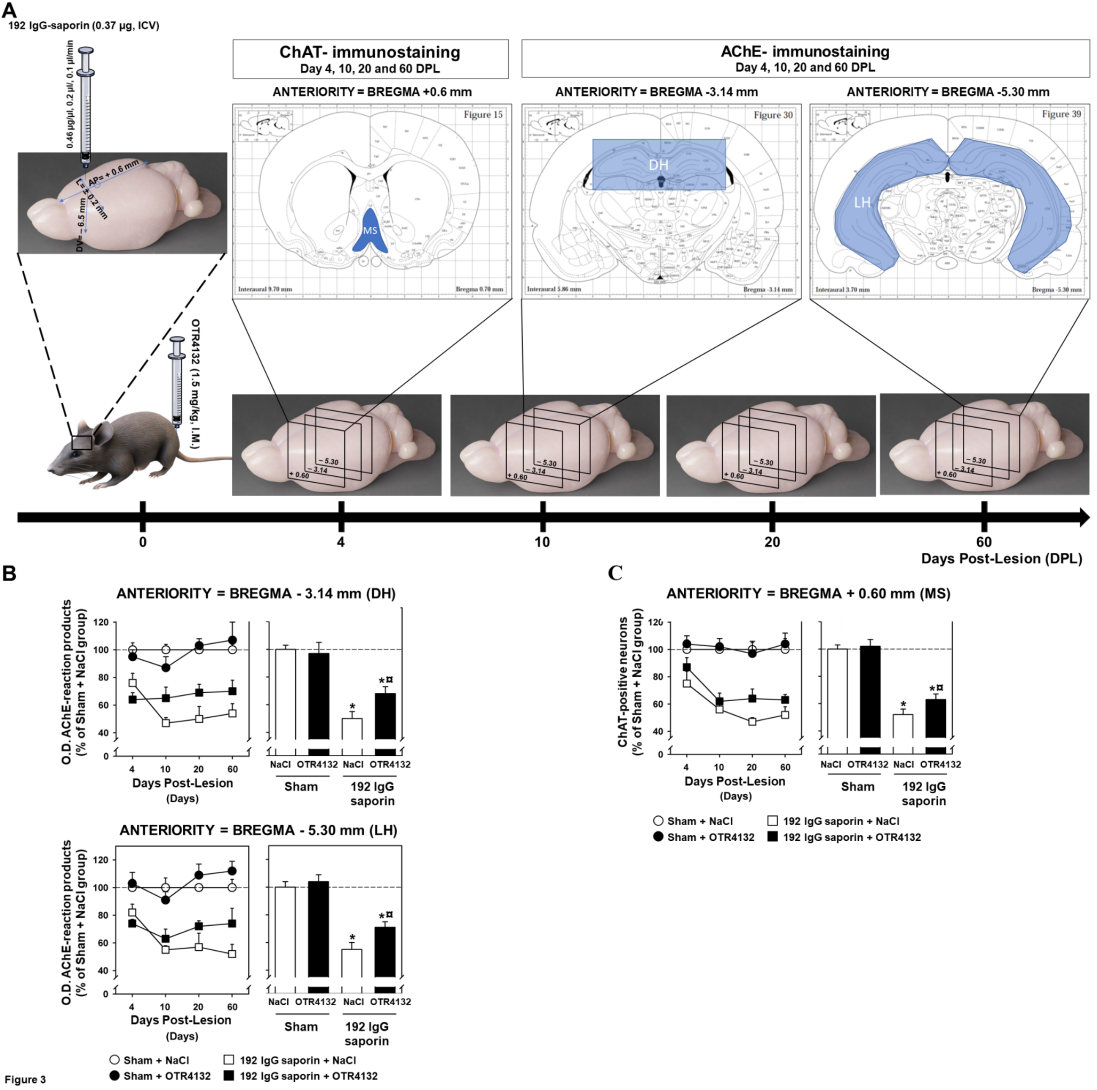
**Effects of I.M. OTR4132 administration after partial lesion of septal cholinergic neurons.** (**A**) Illustration of the experimental design. Rats were first either injected with aCSF or 192 IgG saporin (0.37 µg, ICV), and then they received I.M. injection of OTR4132 (1.5 mg/kg). Brains were harvested 4, 10, 20 and 60 days post-lesion (DPL) and ChAT- and AChE-immunostainings were performed at the medial septum (+ 0.6 mm) and hippocampus sections of each brain (-3.14 mm and -5.30 mm) according to the brain rat stereotaxic coordinates [36]. (**B**) Quantification of brain-immunostaining by optical density (O.D.) assessment of AChE-positive neurons in coronal brain sections through the dorsal hippocampus at an anterior (AP= -3.14 mm; top panels) or posterior (AP= -5.30 mm; bottom panels) levels. Data from (left panels) collapsed for each group for the anterior or the posterior (right panels) levels (**C**) Quantification of brain-immunostaining by optical density (O.D.) assessment of ChAT-positive neurons in coronal brain sections medial septum (AP= +0.6 mm,) in the same groups of rats as in (**A**), including rats with sham surgery injected immediately by I.M. with saline (NaCl 0.9%) or OTR4132 (1.5 mg/kg) solutions, or rats with 192 IgG-saporin lesion followed immediately by I.M. injection of saline or OTR4132 (1.5 mg/kg) solutions. Data from (left panels) collapsed for each group for the data from medial septum (right panels) levels. Data are represented as percentages of the mean O.D. ± SD compared to Sham + NaCl group collected at day 4, 10, 20, and 60 post-lesion The number of rats per group is from three to four (n= 3-4). * Indicates a significant effect of the lesion compared to Sham groups, *p-value*< 0.05, ANOVA test. ¤ Indicates a significant effect of I.M. injection of OTR4132 compared to saporin group treated with NaCl, *p-value*< 0.05, based on ANOVA-test followed by post-hoc Tukey test. AChE: acetylcholinesterase; ChAT: choline acetyltransferase; DH: dorsal hippocampus; LH: lateral part of the hippocampus; MS: medial septum.

Based on the data hereinbefore, to further develop potential therapeutic approaches, OTR4132 was administrated I.M. at 1.5 mg/kg in rats to test its protection on cholinergic neurons from partial lesions induced by 192 IgG-saporin (Fig. 3A). As in the previous experiment, hippocampal slices were stained for AChE-positive fibers and medial septal slices for ChAT-positive neurons. The overall histological findings were similar to those observed after OTR4132 ICV injection, in terms of both the density of AChE reaction products in the hippocampus and the number of ChAT-positive neurons in the MS. Thus, I.M. administration of OTR4132 at 1.5 mg/kg induced a significant 20 to 30% increase in the O.D. of AChE reaction products (measured at both hippocampal levels i.e. AP= -3.14 mm and AP= **-**5.30 mm; Fig. 3B) in lesioned rats compared to untreated lesioned rats (i.e. 192 IgG saporin + NaCl 0.9%). At the anterior level (AP= -3.14; Fig. 3B, top panels), from 10 DPL onward, ANOVA showed significant effects of Group (F 1/56= 85.7, *p-value*< 0.001, ANOVA-test) and Drug (F 1/56= 4.14, *p-value*< 0.05, ANOVA-test), as well as a significant Group-Drug interaction (F 1/56= 6.4, *p-value*< 0.05, ANOVA-test). This interaction was ascribable to a significantly higher O.D. value in the OTR4132-treated lesioned rats compared to their saline-treated lesioned counterparts. No such difference was found in the sham-operated animals. There was no significant DPL effect (F 2/56= 1.3) and no other significant interaction was found (F 2/56< 1.0).

Moreover, at a more posterior hippocampal level (AP= -5.30 mm; Fig. 3B, bottom panels), significant effects of Group (F 1/56= 121.2, *p-value*< 0.001, ANOVA-test) and Drug (F 1/56= 6.148, *p-value*< 0.05, ANOVA-test) were observed, with an overall increase in the O.D. of AChE reaction products induced by the I.M. OTR4132 treatment.

Similarly, OTR4132 I.M. injection significantly increased ChAT-positive neuron counts in the septal area after lesion induction at the various anteriority levels, compared to saline injection (Fig. 3C, left panel, DPL= 20). However, this increase was not but close to significancy (*p-value*= 0.058, ANOVA-test followed by post-hoc Tukey test; Fig. 3C, right panel).

Taken together, these results indicate that, after I.M. injection, OTR4132 is able to significantly protect cholinergic neurons at the medial septum projecting to the hippocampus from partial lesions induced by specific 192 IgG saporin, and thus OTR4132 is able to reach the injured area of the brain.

### I.M. administration of fluorescent OTR4132 reaches hippocampal injuries

**Fig. 4:**
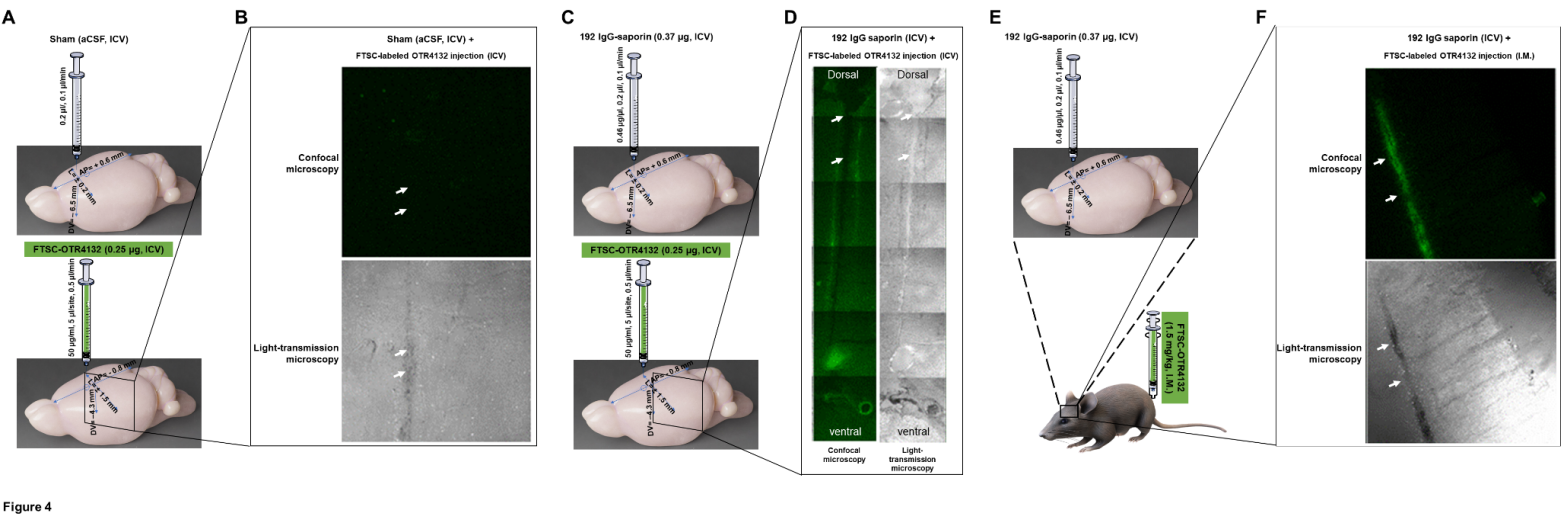
**Confocal microscopic detection of fluorescent OTR4132 injected ICV or I.M. in the septal region**. (**A**) Illustration of the experimental design. Rats were injected with aCSF (ICV) and then injected by fluorescent labelled FTSC-OTR4132 (0.25 µg, ICV). (**B**) Representative of confocal microscopy (upper panel) or light transmission microscopy photomicrographs (lower panel of coronal cryostat sections through the septal region of rats subjected to a sham surgery from (**A**). (**C**) Rats were injected with 192 IgG-saporin (0.37 µg ICV) and then injected by FTSC-OTR4132 (0.25 µg, ICV). (**D**) The cannula track can be identified in both confocal microscopy (left panel) and light transmission microscopy (right panel) photomicrographs, and pinpointed by white arrows. In photographs (left panels), the ventral part of the septum is on the bottom. (**E**) Rats were injected with 192 IgG-saporin (0.37 µg ICV) and then they received I.M. injection of FTSC-OTR4132 (1.5 mg/kg). (**F**) Green fluorescent signal is from FTSC-labeled OTR4132 after I.M. administration at the injection site of 192 IgG-saporin lesion in the medial septum identified in both confocal microscopy (left panel) and light transmission microscopy (right panel) photomicrographs, and pinpointed by white arrows. FTSC: fluorescein-5- thiosemicarbazide. Number of rats for Sham + ICV FTSC-labeled OTR4132 injection, n= 2; 192 IgG-saporin + I.M. FTSC-labeled OTR4132 injection, n=3, and 192 IgG-saporin + ICV FTSC-labeled OTR4132 injection, n=2).

To check that I.M. injection of OTR4132 indeed can cross the BBB and penetrate into the brain to specifically reach the partial lesions of cholinergic neurons **(**Fig. 4**)**, rats received intracerebral injection of aCSF **(**Fig. 4A**)** or 192 IgG-saporin **(**Fig. 4C**)**, and immediately after, received ICV **(**Fig. 4C**)** or I.M. injections **(**Fig. 4E**)** of FTSC-labeled OTR4132. Furthermore, to determine the duration of OTR4132 persistence at the lesion site, rats injected with FTSC-OTR4132 were killed 4, 10, and 20 DPL to be analyzed.

As shown in Fig. 4, a fluorescent signal from FTSC-labeled OTR4132 was identified in the MS at all the DPLs studied. Interestingly, this signal was detected after both ICV (Fig. 4B and D) or I.M. injection (Fig. 4F) of OTR4132. Also, the fluorescent signal was confined to the lesion site and no fluorescence was noticed in the hippocampus or other brain regions. There was no fluorescence in brains from sham-operated rats given intraseptal saline instead of 192 IgG-saporin.

These results indicate that OTR4132 is able to reach and stay only in the injured area of the brain even when I.M. injected.

Taken together with the results from Fig. 3, the data indicate that OTR4132 injected I.M. is able to reach the injured area of the brain, in agreement with the fluorescence observed in the lesioned region of the brain after injection of OTR4132-FTSC.

### Effects of heparin administration after partial lesion to the septal cholinergic neurons

**Fig. 5:**
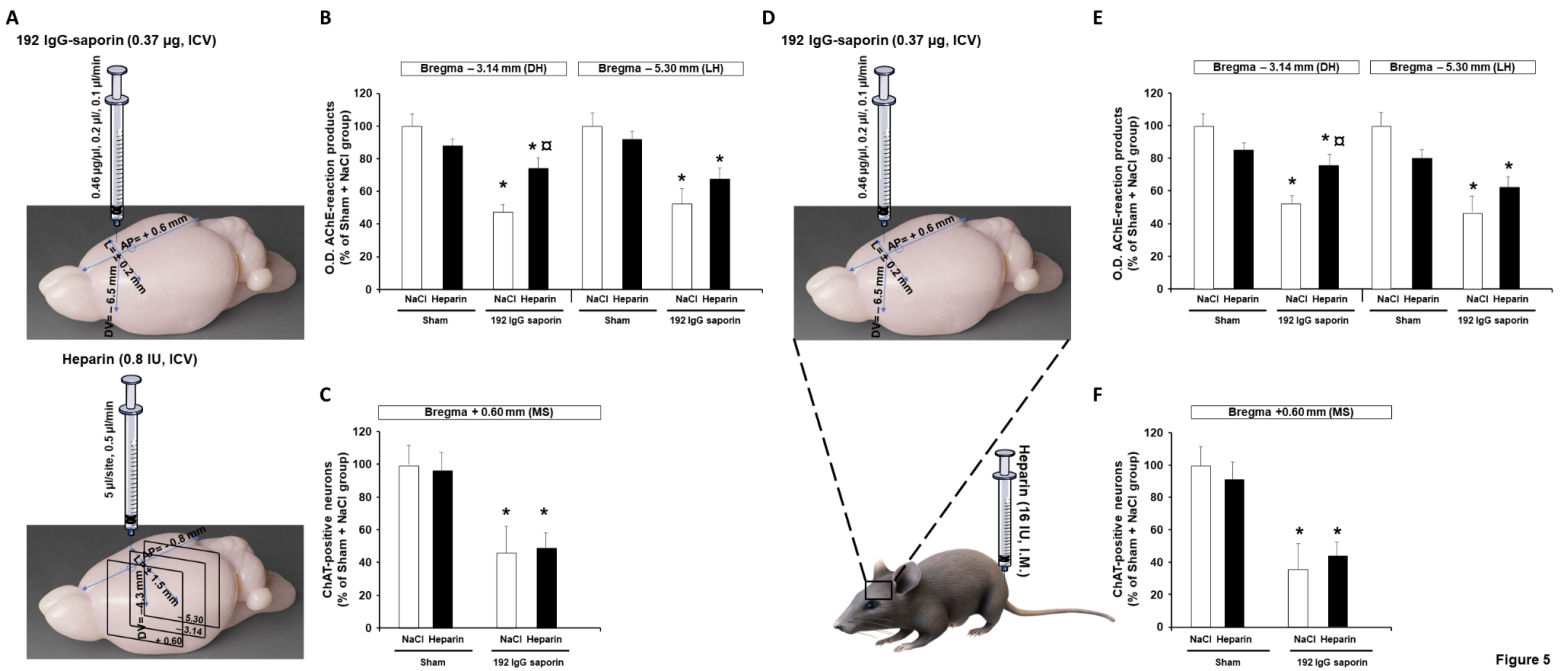
**Effects of ICV or I.M. heparin injection after partial lesion of the septal cholinergic neurons.** (**A**) Illustration of the experimental design. Rats were first either injected with aCSF or 192 IgG saporin (0.37 µg, ICV), and then injected with salin or heparin (0.8 IU, ICV). Brains were harvested 20 days post-lesion (DPL) and ChAT- and AChE-immunostainings were performed at the medial septum (MS, + 0.6 mm) and hippocampus sections of each brain (-3.14 mm and -5.30 mm) according to the brain rat stereotaxic coordinates [36]. Quantification of brain-immunostaining by optical density (O.D.) assessment of (**B**) AChE-positive neurons in coronal brain sections through the dorsal hippocampus at an anterior (AP= -3.14 mm) or posterior (AP= -5.30 mm), and (**C**) ChAT-positive neurons in coronal brain sections of medial septum (AP= +0.6 mm) after ICV injection of heparin at 0.8 UI/ml. (**D**) Another groups of rats were first either injected with aCSF or 192 IgG saporin (0.37 µg, ICV), and then injected by I.M. with saline or heparin (16 IU). Brains were harvested 20 days post-lesion (DPL), and AChE- and ChAT-immunostainings were performed at (**E**) hippocampal sections (-3.14 mm and -5.30 mm) and (**F**) the medial septum (MS, + 0.6 mm) for each brain, and the optical density (O.D.) was measured. Data are percentages of O.D. values compared to control (sham-operated animals given NaCl). The number of rats per group range from 3 to 4 (n= 3/4). Data are represented as mean ± SD. * significantly different from Sham + NaCl. p-value< 0.05. ¤ significantly different from Lesion (i.e. 192 IgG saporin + NaCl). *p-value*< 0.05. Two-way ANOVA. AChE: acetylcholine esterase; ChAT: choline acetyltransferase.

RGTA® such as OTR4132 have been specifically designed to be structurally similar to heparin and heparan sulfates to potentially replace them, as future treatments after brain lesions such as in AD [23, 24, 56]. Thus, effects of heparin administrated by ICV or I.M. injections have been tested in the model of partial lesions of cholinergic neurons induced by intracerebral injection of 192 IgG-saporin (Fig. 5).

The data showed that ICV or I.M. injection of heparin (Fig. 5A and D, respectively) has significant protective effects, similar to those observed with OTR4132 in the hippocampal anterior level (AP= -3.14 mm), but not at the posterior (AP= -5.30 mm) level (Fig. 5B, left and right panels, respectively**)**. At the anterior level (AP= -3.14 mm, Fig. 5B and E, left panel), acetylcholinesterase staining in hippocampal slices showed a significant Group effect (ICV: F 1/21=28.91; I.M.: F 1/19=34.156; *p-value*< 0.001; ANOVA-test), no significant Drug effect (ICV: F 1/21=1.0; *p-value*= 0.33; I.M.: F 1/19 <1.0; ANOVA-test), and a significant Group-Drug interaction (ICV: F 1/21=12.34, *p-value*< 0.05; I.M.: F 1/19=15.41, *p-value*< 0.001, ANOVA-test). Our data show that the Group-by-Drug interaction was ascribable to significantly higher O.D. values in heparin-treated lesioned rats compared to their saline-treated counterparts (*p-value*< 0.001, ANOVA-test). The difference between heparin and saline-treatment was not significant in sham-operated rats (data are summarized in Fig. 5B and E).

At the posterior level (AP= -5.30 mm), acetylcholinesterase staining in hippocampal slices also showed a significant Group effect (ICV: F 1/21=24.36; I.M.: F 1/19=46.14; *p-value*< 0.001, ANOVA-test) but no significant Drug effect (ICV: F 1/21<1.0; I.M.: F 1/19 <1.0). The “Group x Drug” interaction was significant with I.M. administration (F 1/19= 10.82, *p-value*< 0.05, ANOVA-test) but not with ICV administration (F 1/21= 2.5; *p-value*= 0.13, ANOVA-test; data are summarized in Fig. 5B and E). The significant interaction was ascribable to the higher O.D. values in lesioned rats treated with heparin, but not in their sham-operated counterparts.

Then, the effects of ICV or I.M. injection of heparin in ChAT-positive neurons by immunostaining in the medial septum and vertical limb of the diagonal band of Broca (Bregma +0.6 mm; Fig. 5C and F) had no effect on the survival of ChAT-positive.

Indeed, the results showed a Group effect (ICV: F1/21= 19.28; I.M.: F 1/17= 53.12; *p-value*< 0.001, ANOVA-test) but no significant Drug effect (F 1/21 and F 1/17< 1.0) and no significant “Group x Drug” interaction (F 1/21< 1.0; F 1/17= 1.19, *p-value*= 0.29). The Group effect reflected a significant 50–60% reduction in the number of septal ChAT-positive neurons in both lesion groups (*p-value*< 0.001, ANOVA-test, Fig. 5 and F).

Taken together, these results indicate that heparin is less efficient than OTR4132 in protecting cholinergic neurons from 192 IgG-saporin induced partial injury.

### OTR4132 I.M. administration does not protect cholinergic neurons from larger septal lesions

**Fig. 6:**
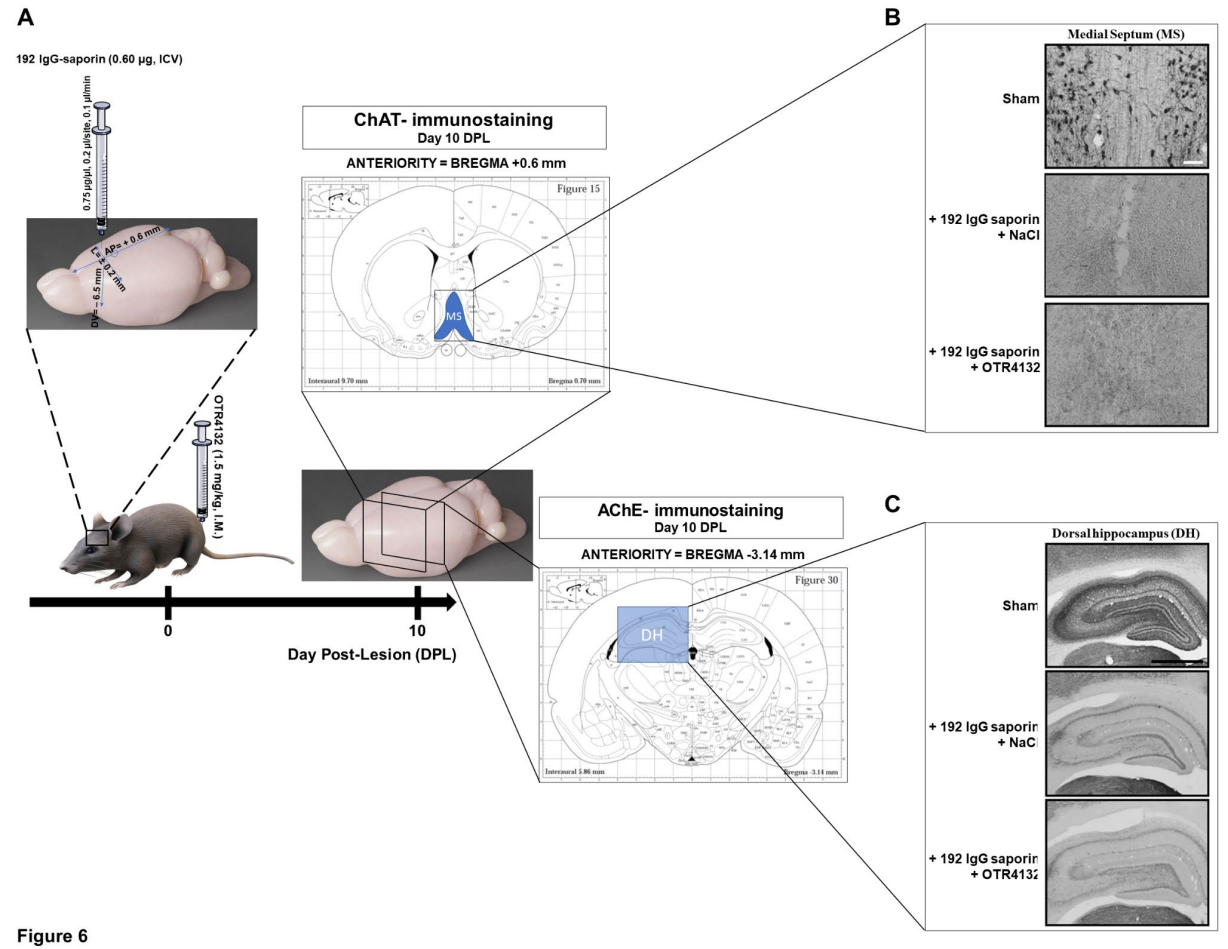
**Effects of I.M. OTR4132 administration after extensive lesion of septal cholinergic neurons.** (**A**) Illustration of the experimental design. Rats were first either injected with aCSF or 192 IgG saporin (0.60 µg, ICV) to induce extensive lesion of septal cholinergic neurons, and then either injected by I.M. with saline (0.9%) or by OTR4132 (1.5 mg/kg). Brains were harvested 10 days post-lesion (DPL) and ChAT- and AChE-immunostainings were performed at the medial septum (+ 0.6 mm) and hippocampus sections of each brain (-3.14 mm) according to the brain rat stereotaxic coordinates [36]. Representative photomicrographs of coronal cryostat sections through the (**B**) dorsal hippocampus or (**C**) medial septum of rats subjected to a sham surgery (top panels), or injected with high-dose (0.60 µg) of 192 IgG-saporin followed immediately by I.M. with saline (middle panels), or by I.M. with OTR4132 (bottom panels). Number of rats per group was three (n=3). Black scale bar= 2.0 mm; and white scale bar= 50 μm. DH: dorsal hippocampus; MS: medial septum.

In Fig. 3, data showed that I.M. injection of OTR4132 significantly protects cholinergic neurons from partial septal lesion. To go further with OTR4132 neuroprotection effects, we explored a possible neuroprotective/neurotrophic effect of I.M. injected OTR4132 in animals with larger lesions in cholinergic neurons. Rats were given a higher dose (0.6 µg) of 192 IgG-saporin, immediately followed by I.M. OTR4132 injection (1.5 mg/kg; *n*= 3 under each lesion and drug condition). As illustrated in Fig. 6A, at 10 DPL, no ChAT-positive neurons in the septal (Fig. 6B) or AChE reaction products in the hippocampus (Fig. 6C) area were detected following the administration of the higher dose of 192 IgG-saporin (0.60 µg). Under these conditions, no beneficial effect of OTR4132 were detectable.

Taken together, these results show that OTR4132 protects cholinergic neurons of the medial septum projecting to the hippocampus from partial, but not extensive, lesions. These data suggest that OTR4132 treament has to be administered early during the development of cholinergic neuron degenerescence to be effective.

### Intraseptal co-injection of OTR4132 and fluorescent 192 IgG

**Fig. 7:**
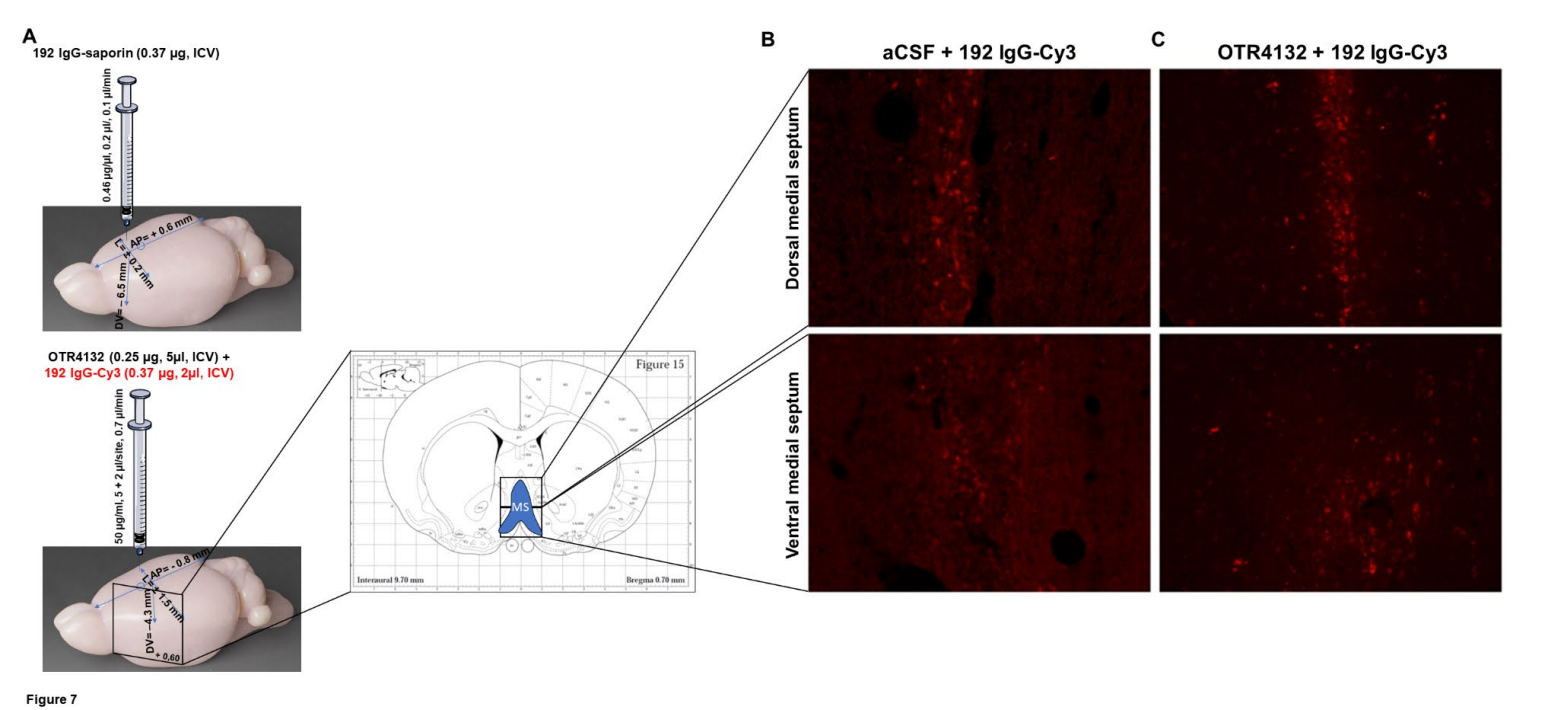
**Intraseptal co-injection of OTR4132 and fluorescent 192 IgG.** (**A**) Illustration of the experimental design. Rats were first injected with 192 IgG saporin (0.37 µg, ICV), and then injected with 192 IgG-Cy3 alone (37 µg, ICV) or co-injected with OTR4132 (0.25 µg, ICV). Brains were harvested 10 days post-lesion (DPL) and immunoflurorescenvce performed at the medial septum (+ 0.6 mm) according to the brain rat stereotaxic coordinates [36]. Representative immunofluorescence photomicrographs of cryostat sections of intraseptal injection of (**B**) 192 IgG-Cy3 alone or (**C**) co-injected with OTR4132 through the dorsal (top panels) or the ventral (bottom panels) medial septum in rats (n= 3). MS: medial septum.

To evaluate whether nonspecific binding of OTR4132 to p75-NTR receptor might prevent the binding and internalization of 192 IgG-saporin, thus explaining the decrease in lesion severity after OTR4132 treatment, we assessed the internalization of fluorescent 192 Cy3-labeled IgG by confocal microscopy after intraseptal co-injection or not of OTR4132 (Fig. 7A). Results in Fig. 7B and C showed that no difference in fluorescence staining was observed between rats that received or not OTR4132 (Fig. 7B and C), both at a dorsal (top panels) and at a ventral level of the medial septum (bottom panels). Accordingly, the quantitative fluorescence analyses of the Cy3 positive neurons did not show significant differences between the group of rats receiving 192 IgG-Cy3 alone (Fig. 7B) and those co-injected with 192 IgG-Cy3 + OTR4132 (Fig. 7C).

These results show that OTR4132 does not prevent the internalization of fluorescent 192 Cy3-labeled IgG, and thus OTR4132 decreases the severity of the MS lesions of 192 IgG-saporin by a mechanism different from competing with the mechanisms usually involved in internalization of 192 IgG-saporin into the neurons, which is based on IgG specific interaction with target protein on the cell surface [57]. Also, 192 IgG-saporin is a protein composed of around 250-290 amino acids, corresponding to a molecular weight of 28.6 kDa with alpha-folds, it does not contain an heparin binding-site nor a cluster of basic amino acids (D,R,E, K and/or H). Thus, it is unlikely that OTR4132 can directly bind to 192 IgG-saporin.

### Effect of OTR4132 in the accumulation of [³H]choline in hippocampal slices and baseline outflow of [³H]

**Fig. 8:**
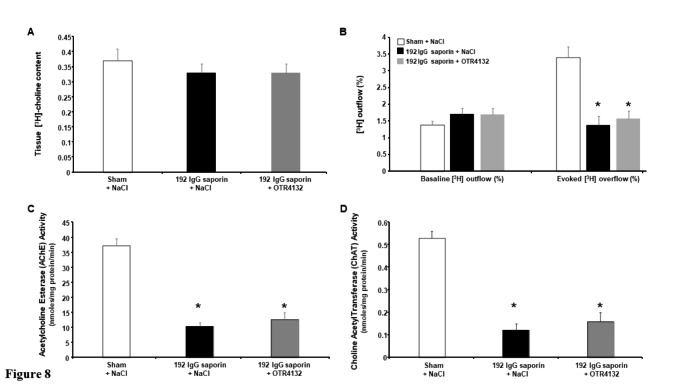
**Effect of OTR4132 in the accumulation of [³H]-choline in hippocampal slices and baseline outflow of [³H]. (A)** [^3^ H] accumulation, (**B**) basal [^3^ H] outflow, and electrically-evoked [^3^ H] overflow in hippocampal slices (in % of [^3^ H] uptake] preincubated with [^3^ H]-choline, (**C**) acetylcholine esterase and (**D**) choline acetyltransferase activities (in nmoles/mg protein/min) from rats subjected to a sham surgery, 192 IgG-saporin injection, or 192 IgG-saporin injection followed immediately by OTR4132 treatment. Data are means ± s.e.m, n= 3 rats per group. *significantly different from Sham + NaCl group, p-value< 0.05, ANOVA-test follow by post-hoc Tukey test. AChE: acetylcholinesterase; ChAT: choline acetyltransferase.

We further assessed the effect of OTR4132 on tritiated choline ([³H]-choline) accumulation and on baseline outflow of [³H] in hippocampal slices preincubated with [³H]choline, expressed as the percentage (%) of choline uptake (Fig. 8). Whereas no effect was observed in [³H]choline accumulation (Drug effect: F 2/19= 1.27, N.S., ANOVA-test; Group effect: F 2/19< 1.0, NS, ANOVA-test, Fig. 8 A) and as expected on baseline [^3^ H] outflow (Fig. 8B), a significant Group effect was observed in the [³H] overflow, which corresponds to the overflow of ³[H] in response to the first electrical field stimulation (S_1_) reported as the percentage of tissue-[³H] (Fig. 8B, Evoked [^3^ H] outflow, F 2/19= 16.7; p-value< 0.001, ANOVA-test). However, no effect was observed for OTR4132-treated lesioned rats (192 IgG-saporin + OTR4132) compared to the saline-treated lesioned rats (192 IgG-saporin + NaCl) (Fig. 8B, Evoked [^3^ H] outflow, non-significant post-hoc Turkey test).

Moreover, acetylcholine esterase (AChE) and choline acetyltransferase (ChAT) activities were assessed in the ventral hippocampus homogenates. In lesioned rats treated with saline (192 IgG-saporin + NaCl) compared to the Sham group, both enzymatic activities were significantly reduced by about 70% (ChAT: F 2/19= 52.55, *p-value*< 0.001, ANOVA-test; and AChE: F 2/19= 48.61; *p-value*< 0.001, ANOVA-test, Fig. 8C and D, respectively). However, under these conditions, the lesioned rats treated with OTR4132 (192 IgG-saporin + OTR4132) were not significantly different from those treated with saline (192 IgG-saporin + NaCl, Fig. 8C and D).

**Fig. 9:**
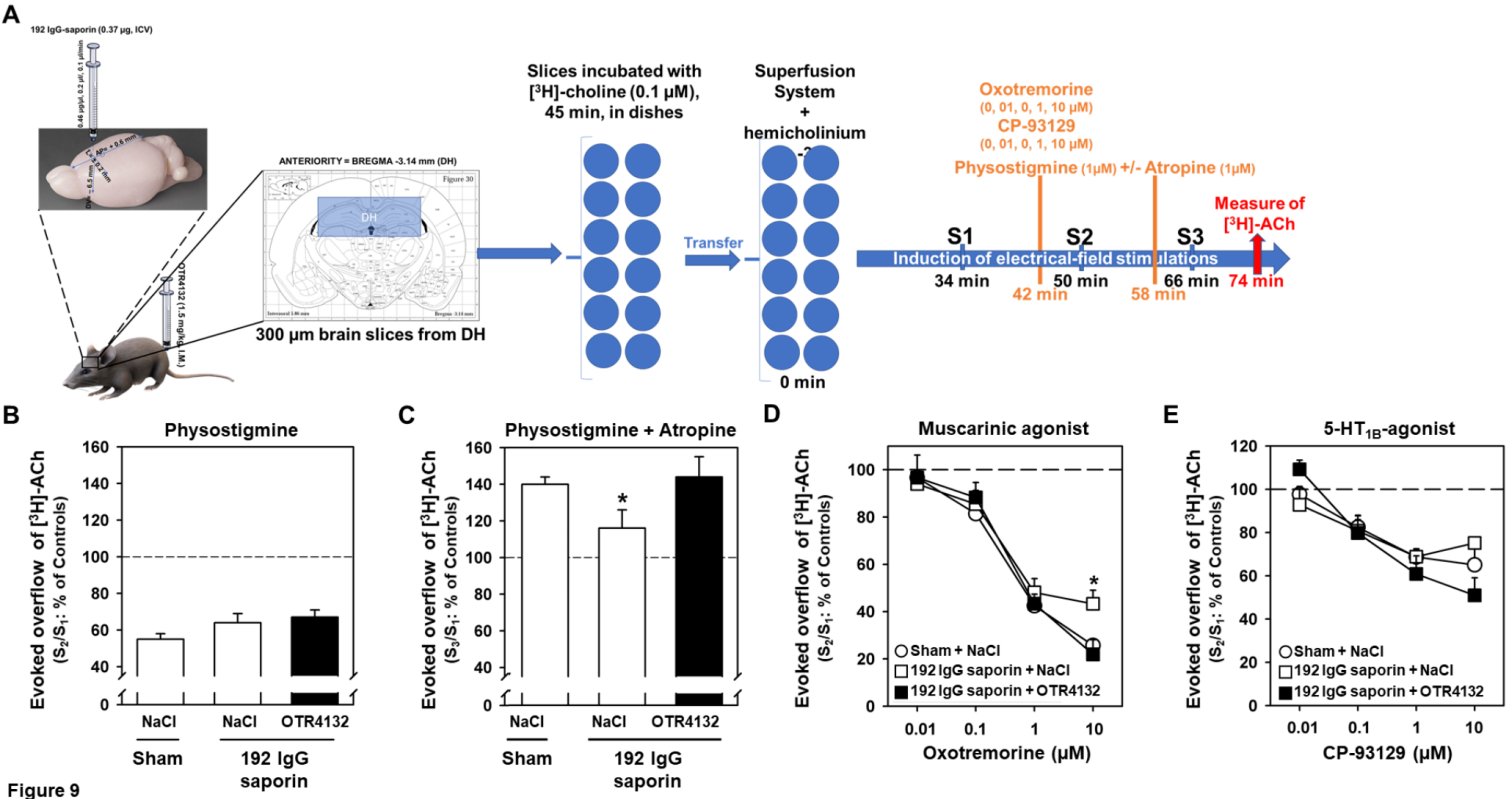
**OTR4132 protecting effects and potential physiological effects on cholinergic neurons affected by 192 IgG-saporin using pharmacological approaches.** (**A**) Illustration of the experimental design. Rats were first injected with 192 IgG saporin (0.37 µg, ICV), and then injected with OTR4132 (1.5 mg/kg, I.M.). Coronal slices (300 μm thick) of the dorsal hippocampus were prepared and incubated with [^3^ H]-choline (0.1 µM) during 45 min at 37 °C. Then, slices were transferred into superfusion chambers (12 chambers per superfusion apparatus, 1 slice per chamber) and superfused with oxygenated KH buffer and supplemented with hemicholinium-3 (10 µM). A first electrical field stimulation was applied 15 min after the start of the superfusion. Fractions were collected from 32 min of superfusion onward. [^3^ H]-ACh release was induced by up to three electrical-field stimulations, after 34 min (S1), 50 min (S2), and 66 min (S3) of superfusion. Drugs to be tested were added to the superfusion medium of some chambers from 8 min before S2 and S3 onward at different concentrations as followed. Effects of (**B**) physostigmine (1 µM) alone or (**C**) with atropine (1 µM) on electrically-evoked [^3^ H]-ACh release from hippocampal slices of rats subjected to a sham surgery or 192 IgG-saporin injection followed immediately by I.M. injection of NaCl 0.9% solution (NaCl) or OTR4132 solution. Data are represented as mean ± SD. * *p-value*< 0.05 compared to Sham and 192 IgG-saporin + OTR4132 groups, ANOVA-test followed by post-hoc Tukey test. (**D**) Effect of the muscarinic receptor agonist oxotremorine on electrically-evoked [^3^ H]-ACh release from hippocampal slices of rats subjected to a sham surgery 192 IgG-saporin injection (192 IgG saporin + NaCl), or 192 IgG-saporin followed immediately by I.M. OTR4132 (192 IgG saporin + NaCl). Data are represented as mean ± SD. * *p-value*< 0.05; ANOVA-test) compared to both Sham, 192 IgG saporin + NaCl and 192 IgG saporin + OTR4132 groups. (**E**) Effects of OTR4132 on serotonin function based on electrically-evoked overflow of [^3^ H]-ACh in hippocampal slices preincubated with [³H]choline. Brain slices were subjected to 5-HT_1B_ receptor agonist CP-93,129 on electrically-evoked [^3^ H]-ACh release from hippocampal slices of rats subjected to a sham surgery (Sham + NaCl), 192 IgG-saporin injection followed immediately by I.M. injection of NaCl (192 IgG saporin + NaCl) or OTR4132 (192 IgG saporin + OTR4132). Data are the mean ± SD and analysed by ANOVA-test. The number of rats per group ranged from three to four (n= 3-4). ACh: acetylcholine.

To go further on understanding OTR4132 protecting effects and potential physiological effects on cholinergic neurons affected by 192 IgG-saporin, pharmacological approaches have been used (Fig. 9A). To assess if OTR4132 modified evoked overflow of [^3^ H]-ACh, physostigmine was used alone or associated with atropine. Previous studies have shown that physostigmine alone can reduce the evoked [³H] overflow [33, 58]. Accordingly, in this study, the results showed that physostigmine significantly reduced by about 45% the [³H] overflow in hippocampal slices preincubated with [³H]-ACh (Fig. 9B), its inhibitory effects being slightly (but not significantly) less pronounced in lesioned animal treated with NaCl or with OTR4132 (35% and 30%, respectively, Fig. 9B).

Moreover, the simultaneous presence of atropine not only abolished this reduction, but also increased the evoked [³H]-ACh overflow by up to 33% (Fig. 9C). Statistical analyses revealed a significant Drug effect (F 1/18= 210.2, *p-value*< 0.001, ANOVA-test) and a significant “Group x Drug” interaction (F2/18= 1.78: *p-value*< 0.05, ANOVA-test), but not significant Group effect (F2/18= 3.51; *p-value*= 0.52, ANOVA-test). The 16% increase in the evoked overflow of [³H]-ACh mediated in the association physostigmine and atropine in lesioned rats treated with NaCl (+ 192 IgG saporin + NaCl) was significantly lower (Fig. 9C, p*-value*< 0.05, ANOVA-test) compared to both sham-operated rats and lesioned rats treated with OTR4132 (+ 192 IgG saporin + OTR4132), for which the increase was 38% and 45%, respectively.

To get more mechanistic insights, the effects of the muscarinic agonist oxotremorine on electrically-evoked overflow of [^3^ H] in hippocampal slices preincubated with [³H]-choline was assessed in animals treated or not with OTR4132 (Fig. 9D). The data showed that increasing oxotremorine doses significantly inhibited the electrically-evoked [³H] overflow (dose-effect: F(3/42)= 235.93; *p-value*< 0.001, ANOVA-test). We additionally observed a significant Group effect (F(2/14)= 7.71; *p-value<* 0.05, ANOVA-test), with a significantly smaller decrease of the release in lesioned rats given saline (192 IgG saporin + NaCl) compared to sham-operated rats (Sham + NaCl) or OTR4132-treated lesioned rats (192 IgG saporin + OTR4132; *p-value*< 0.01, ANOVA-test followed by pos-hoc tukey test). The effects of oxotremorine on the evoked [³H] overflow in sham-operated rats (Sham + NaCl) were not different from those in OTR4132-treated lesioned rats (192 IgG saporin + OTR4132; *p-value=* 0.53, Fig. 9D). The results also showed a significant interaction between Dose and Group comparison (F(6/42)= 14.61; *p-value*< 0.001; ANOVA-tets), especially with the highest dose of oxotremorine (10 µM), which induced a larger decrease in the evoked release in the sham-operated (Sham + NaCl, -75%) and lesioned rats treated with OTR4132 (192 IgG saporin + OTR4132, -79%) than in the lesioned rats given saline (192 IgG saporin + NaCl, -57%; *p-value*< 0.001; ANOVA-test followed by pos-hoc tukey test, Fig. 9D). These results suggest that OTR4132 failed to reverse the decrease in electrically-evoked [^3^ H]-ACh release during the first stimulation period (S_1_). However, OTR4132 attenuated the lesion effects on the responsiveness of inhibitory muscarinic autoreceptors to oxotremorine.

Then, the effect of OTR4132 was tested on the 5-HT_1B_ signaling pathway by using a selective agonist, CP-93,129, on electrically-evoked overflow of [^3^ H]-ACh in hippocampal slices preincubated with [³H]choline (Fig. 9E). The results showed that the inhibitory effects of the 5-HT_1B_ agonist CP-93,129 increased in a dose dependent manner in the Sham group, as expected. Indeed, the evoked [³H]-ACh overflow significantly decreased when CP-93,129 concentrations increased (Fig. 9E, Sham + NaCl Dose-effect: F(3/39)= 23.12; *p-value*< 0.001, ANOVA-test). The evoked [³H]-ACh overflow also significantly decreased in brain slices of rats from 192 IgG saporin + NaCl and 192 IgG saporin + OTR4132 groups when CP-93,129 concentrations increased (Fig. 9E, p*-value*< 0.001, ANOVA-test), whereas there was neither a significant Group effect (F(2/13) *p-value*< 1.0, ANOVA-test) nor a significant “Group x Drug” interaction (F(6/39)= 2.02; *p-value*= 0.08, ANOVA-test), indicating that neither the lesion nor OTR4132 significantly modified the effects of CP-93,129 (Fig. 9E).

Taken together, these data showed that I.M. administration of OTR4132 attenuated the decrease of muscarinic auto-receptor sensitivity after 192 IgG-saporin lesions, but such lesions, whether combined with OTR4132 treatment or not, did not produce changes in serotoninergic heteroreceptor sensitivity. This suggests that, although the decrease in electrically-evoked [^3^ H]-ACh release during the first stimulation period (S_1_) was slightly but not significantly improved by OTR4132, the mimetic was able to attenuate the lesion effects by acting on the responsiveness of inhibitory muscarinic autoreceptors to oxotremorine without altering the presynaptic serotoninergic modulation of [^3^ H]-ACh release.

## Discussion

During the last decades, it has been shown that basal forebrain cholinergic neurons undergo degeneration in some brain diseases, such as Alzheimer’s disease, stroke, and traumatic brain injury [59–61]. Therefore, compounds that protect neurons from degeneration are candidates for potential therapeutic intervention [62]. In this study, by using a model of partial immunotoxic lesion of septal cholinergic neurons induced by 192 IgG-sapo injection, we assessed the capacity of the glycosaminoglycan mimetic named OTR4132 to protect cholinergic neurons from degeneration. We first showed that OTR4132 administered within the lateral ventricles (i.e. ICV) or by I.M. injection significantly protect MS cholinergic neurons and their projection fibers in the hippocampus. Accordingly, OTR4132 significantly restored the function of hippocampal muscarinic autoreceptors on hippocampal cholinergic terminals in the mice model. By using a fluorescent OTR4132, we showed that OTR4132-FTSC administered I.M. could accumulate in the injured brain region, suggesting that, in 192 IgG-saporin-induced brain injury, the compound could cross the BBB. In the first part of our study, we validated the model using small amounts (0.37 µg) of 192 IgG-saporin to induce a selective but partial lesion of the septohippocampal cholinergic pathways, as previously described [31, 47, 48]. Accordingly, septal GABAergic neurons expressing parvalbumin were relatively preserved. The model was characterized by the loss of about 50% of septal cholinergic neurons near the immunotoxin injection sites (i.e. at the level of the cannula tracks). In the OTR4132 non-treated injured dorsal hippocampus, the density of AChE reaction products decreased by about 45–50%, at both the anterior and the posterior levels. It is well known that the intensity of AChE staining in the hippocampus reflects the density of cholinergic septohippocampal inputs [63, 64]. Accordingly, the lesion in our model probably deprived the hippocampus of approximately 50% of its cholinergic inputs. Interestingly, the toxic effect of 192 IgG-saporin was the greatest at about 10 days after the neurotoxin injection, and remained stable thereafter until our latest time point (i.e.60 DPL). Previous reports found that ChAT activity depletion in brain regions innervated by basal forebrain cholinergic neurons was the greatest at about seven days after immunotoxin injection with non spontaneous recovery [65, 66]. After assessing the toxicity of 192 IgG-saporin in forebrain cholinergic neurons, we assessed the neuroprotective effect of OTR4132 administrated ICV or I.M. OTR4132 attenuated the effect of 192 IgG-saporin on septohippocampal cholinergic neurons. This finding is consistent with the reported cholinergic neuroprotective effects of a previously tested glycosaminoglycan analog called GAG-C3 that was not further clinically developed, possibly because of its structural heterogeneity [67, 68]. GAG-C3 was used daily for seven days after cholinergic neurons damage induced by ICV administration of the fairly selective cholinergic toxin named ethylcholine aziridinium (AF64A) [3, 67, 68]. Interestingly, the neuroprotective effects of OTR4132 in the present study occurred after a single OTR4132 injection performed immediately after the immunotoxin injection. In addition, in this study, OTR4132 was found in the lesion site for 20 days after a single administration (as discussed hereinafter). Accordingly, in a recent study, we also showed that OTR4132 injected I.V. protected and repaired the brain after a stroke, favoring functional recovery [23]. Here, we found that OTR4132 not only crossed the BBB, as it was found within the brain after intramuscular injection, but also specifically targeted the lesion site, where it was detectable for at least 20 DPL. Mechanical BBB injury caused by the needle was not responsible for penetration of OTR4132 through the BBB, since no FTSC-labeled OTR4132 was found in the brain of saline-infused control rats. However, we cannot rule out that the immunotoxin itself induced BBB alterations allowing OTR4132 penetration. Roher et al. suggested that injection of 192 IgG saporin into the nucleus basalis of rabbits might result in breaching of the BBB, probably *via* alterations in vascular chemistry [69]. FTSC-labeled OTR4132 was detected in the vicinity of the 192 IgG-saporin-induced lesions. Conceivably, the mechanical injury induced by cannula insertion may result in a milder degree of inflammation when saline is injected, whereas immunotoxin injection may boost the inflammatory response, concentrating specific neurotrophic factors near the lesion site [70]. Thus, the kinetics of local heparanase activity induction may differ between saline and immunotoxin injection, and the number of available chondroitin- and heparan-binding sites in the lesion immediately after the injection may also differ. As FTSC-OTR4132 was injected rapidly after intraseptal insertion of the cannula, there were probably not enough GAG-binding sites available to allow detection in sham animals. Importantly, fluorescent OTR4132 was not identified in the cortex of rats injected with 192 IgG-saporin, although the cannula altered this tissue. Moreover, OTR4132 appeared to selectively target the focus of inflammation produced by the immunotoxin. This site selectivity may limit possible adverse effects on adjacent healthy tissue. Additionally, in a preliminary experiment, no evidence of Evans blue extravasation following intraseptal injection of 192 IgG-saporin was found [2]. Therefore, under our working conditions, the presence of OTR4132 within the lesion reflected the BBB permeability to OTR4132. Selective concentration of RGTA® in and near the lesion has been reported after systemic injection of tritiated RGTA® in a muscle lesion model [71]. RGTA® selectively targeted the muscular lesion and persisted in the lesion for several weeks. As suggested by Meddahi et al., RGTA® would bind specifically to the multiple heparin-binding sites of matrix proteins that become available after lesion-induced degradation of heparan sulfates and chondroitin sulfates [72]. As OTR4132 was administered immediately after 192 IgG-saporin injection, its protective effects may be due to indirect mechanisms. OTR4132 may reduce the internalization of 192 IgG-saporin, and therefore its toxic effects, either by decreasing the penetration of 192 IgG-saporin within cells or by competing with the p75^NTR^ receptors. However, our results obtained with intraseptal co-injection of Cy3-labeled 192 IgG and OTR4132 argue against this possibility. The septal cellular fluorescent signal accounting for saporin incorporation was not different between the rats receiving OTR4132 and their saline-treated counterparts, arguing against milder toxin-induced injury as the mechanism underlying cholinergic neuron protection by OTR4132, but in favor of a direct protective effect of OTR4132 on the extracellular matrix injury induced by 192 IgG-saporin.

Our data suggest that OTR4132 may exert beneficial effects on basal forebrain cholinergic neurons. Although mechanisms involving interaction with growth factors were not studied here, for decades, it was showed that RGTA® interact, protect, and increase the bioavailability of various heparin binding growth factors [9, 21, 71, 73], thus increasing their half-life [9, 21, 74–76]. Conceivably, RGTA® may act *via* mechanisms involving the NGF and/or BDNF-mediated pathways. Accordingly, evidence for neuroprotective effects of growth factors after septohippocampal pathway injury has been reported [12, 77]. Thus, the neuroprotective effects of RGTA® may involve an increase in endogenous neurotrophic factor levels, promoting neuroprotection and repair. This putative mechanism is consistent with the ability of NGF and BDNF to restore impaired cholinergic function in several lesion models [18, 78].A transient lesion-induced increase in NGF alone does not seem sufficient to promote survival and/or recovery in lesioned animals treated with saline only. Treatment with the NGF-protecting OTR4132 may have prolonged the beneficial effects of endogenous NGF, thus supporting the restoration of cholinergic functions.

Previous experiments have shown that highly sulfated glycosaminoglycans, such as chondroitin sulfate E, possess direct neurite growth-promoting activity and reduce the amounts of growth factor needed to promote this activity [79, 80]. Our evaluation of cholinergic marker kinetics suggested that ICV or I.M. injection of OTR4132 may have neuroprotective rather than neurotrophic effects. ChAT immunostaining and histochemically-documented AChE activity, which were similarly reduced at 4 DPL in all lesioned animals, decreased further over time in saline-treated rats but remained stable in their OTR4132-treated counterparts. These findings suggest that OTR4132 slowed and attenuated the lesion-induced decreases in cholinergic markers, rather than promoted restoration after an initially larger reduction. It is noteworthy, however, that the protective effect of OTR4132 varied according to lesion size as it was undetectable in animals with large immunotoxic lesions. The marked septal tissue necrosis seen with the higher toxin amount (Fig. 6B) may have been associated with severe disorganization of the extracellular matrix scaffold, preventing OTR4132 from attaching to the lesion site. Furthermore, the higher toxin concentration may have caused the death of a larger number of cholinergic neurons compared to partial lesions, which may have overwhelmed the rescue/protection capabilities of locally synthesized neurotrophic factors (Fig. 6B).

RGTA® are synthetic heparin analogs that mimic some of the properties of heparin while exhibiting two favorable characteristics, (1) they are not degraded by heparanases, hyaluronidases, or other glycanases, and (2) their anticoagulant activity is only a fraction of that of heparin [22, 37, 77]. Therefore, it was of interest to compare heparin and OTR4132 in our model. In this study, ICV or I.M. injection of heparin had no beneficial effect on the number of cholinergic cell bodies in the MS but attenuated the lesion-induced decrease in hippocampal AChE staining. Thus, unlike OTR4132, heparin failed to protect the septal cholinergic cell bodies but possibly stimulated the sprouting of remaining cholinergic fibers, thereby increasing the AChE activity in the hippocampus. Marked increases in mRNA of heparin-binding growth-associated molecule (HB-GAM) have been reported after hippocampal neuronal injury. HB-GAM protein binds strongly to heparin and enhances neurite outgrowth in vitro [79–81]. The difference in effects between heparin and OTR4132 may be related in part to a higher level of enzyme activity at the site of toxin injection compared to within the hippocampus, leading to a faster degradation of heparin than of OTR4132 in the MS. This fast degradation would limit the effects of heparin to the hippocampal target region, and therefore to neurite outgrowth from the terminals of surviving cells, and would preclude an effect on cell survival. However, we cannot exclude that repeated administrations of heparin with low anticoagulant activity would eventually produce the same effects as those obtained with OTR4132. Also, in a lesion model characterized by a stronger distal inflammatory response compared to our study model, OTR4132 might display a more extensive neurotrophic effect in the CNS compared to heparin. This hypothesis is supported by the recent publication on OTR4132 protection of the brain tissue following stroke injuries [23].

In the present study, some experiments were designed to examine lesion and OTR4132 effects on hippocampal muscarinic autoreceptor functions. Indeed, we assessed the effects of 192 IgG-saporin and OTR4132 on the modulation of electrically-evoked [^3^ H]-ACh release in hippocampal slices. The electrically-evoked overflow of [³H]-ACh from hippocampal slices preincubated with [³H]choline reflects the action potential-induced exocytotic release of [³H]-ACh, as shown previously [47, 48]. A major advantage of the slice superfusion technique is that it enables a focused and reliable investigation of modulation by axon terminals *ex vivo*, using a small piece of brain tissue [47]. Previous studies of the cholinergic neurons originating in the septal area and projecting to the hippocampus showed that acetylcholine release was under inhibitory control involving, among other receptors, muscarinic autoreceptors of the M_2_ type and serotonergic heteroreceptors of the 5-HT_1B_ type [82, 83]. In the present study, OTR4132, failed to induce recovery of the partial lesion-induced decrease in ChAT and AChE activities in the ventral part of the hippocampus (Fig. 8). However, the decrease in electrically-evoked [^3^ H]-ACh release during the first stimulation period (S_1_) was slightly but not significantly improved by OTR4132. In addition, OTR4132 attenuated the lesion effects on the responsiveness of inhibitory muscarinic autoreceptors to oxotremorine (Fig. 9D), without altering the presynaptic serotonergic modulation of [^3^H]-ACh release (Fig. 9E). The level of release in hippocampal slices of saline-treated lesioned rats revealed a decrease in the sensitivity of muscarinic autoreceptors toward both the exogenous agonist oxotremorine and the endogenous agonist ACh. This lesion-induced downregulation of muscarinic autoreceptor sensitivity may ensure sufficient ACh release in the hippocampus by the cholinergic axon terminals spared by the lesion. Similar downregulation of muscarinic autoreceptor sensitivity was, however, not apparent in the rats treated with OTR4132. Thus, administration of the OTR4132 seems either to promote restoration of sensitivity of autoreceptors-mediated inhibition of ACh release in the terminals spared by the lesion or to prevent lesion-induced downregulation of these receptors. Such downregulation was demonstrated in experiments involving more extensive but less selective damage to the cholinergic septohippocampal pathways [82, 83]. Neural growth factors may contribute to the changes in receptor sensitivity. For instance, FGF2 infusion restored neural function in a model of cognitive dysfunction in rat injury induced with Kainic acid, possibly due to mitogenic effect on dentate gyrus neurogenic area leading to generation and migration of newer cholinergic neurons [84]. Under our conditions, OTR4132 treatment may potentiate the beneficial effects of endogenous neurotrophic factors on maintenance of pre-synaptic cholinergic neuronal function, thus preserving/restoring the auto-inhibitory processes impaired by the lesion and the pre-synaptic autoreceptor-mediated regulatory activities. We are currently investigating these hypotheses.

The structure of OTR4132 mimics the highly sulfated glycosaminoglycans such as heparin, heparan sulfates and chondroitin sulfate E, known for their neuroprotective properties. It differs from the structure of the more abundant, weakly sulfated chondroitin sulfates A and B, which inhibit new axonal growth after injury [85]. The current study suggests that potentiation by OTR4132 of the protective effects exerted by growth factors normally bound to extracellular matrix proteoglycans may reduce cholinergic deficits in the degenerating brain. This hypothesis is further supported by studies on the role of altered heparan sulfate structures and functions in the mechanisms leading to Alzheimer’s disease-related [56, 86, 87].

In conclusion, our study shows that OTR4132, a dextran-based polymer, engineered to mimic highly sulfated glycosaminoglycans, may attenuate the extent and functional damages of partial immunotoxic cholinergic lesions. Memory impairments were previously shown to be mild or absent after partial 192 IgG saporin-induced lesions of basal forebrain cholinergic neurons [84, 88, 89]. Therefore, we did not assess effects of OTR4132 on cognition. However, cognitive evaluations, perhaps after the induction of lesions with greater effects on behavior, would be of interest. Our data also show that OTR4132 crossed the likely disrupted BBB, penetrated within lesioned tissues, and persisted in or near the lesion locus. Although the mechanisms of this neuroprotection remain to be elucidated, our findings suggest that OTR4132 may hold promises for the treatment of neurodegenerative disease and perhaps even invasive CNS damage. These CNS protective effects of OTR4132 using multiple injections (e.g. every 2 or 3 days) should be investigated, especially for stroke or AD treatments.

## References

[1] Fidali BC, Stevens RD, Claassen J (2020). Novel approaches to prediction in severe brain injury. Curr. Opin. Neurol..

[2] Marques Pereira P, Cosquer B, Schimchowitsch S, Cassel JC (2005). Hebb-Williams performance and scopolamine challenge in rats with partial immunotoxic hippocampal cholinergic deafferentation. Brain Res. Bull..

[3] Will B, Galani R, Kelche C, Rosenzweig MR (2004). Recovery from brain injury in animals: relative efficacy of environmental enrichment, physical exercise or formal training (1990-2002). Prog Neurobiol..

[4] Zambusi A, Ninkovic J (2020). Regeneration of the central nervous system-principles from brain regeneration in adult zebrafish. World J. Stem Cells.

[5] Boskovic Z, Meier S, Wang Y, Milne MR, Onraet T, Tedoldi A, Coulson EJ (2019). Regulation of cholinergic basal forebrain development, connectivity, and function by neurotrophin receptors. Neuronal Signal..

[6] Isaev NK, Stelmashook EV, Genrikhs EE (2017). Role of Nerve Growth Factor in Plasticity of Forebrain Cholinergic Neurons. Biochem. (Mosc).

[7] Niewiadomska G, Komorowski S, Baksalerska-Pazera M (2002). Amelioration of cholinergic neurons dysfunction in aged rats depends on the continuous supply of NGF. Neurobiol. Aging.

[8] Gustafsson, D., Klang, A., Thams, S., Rostami, E.: The Role of BDNF in Experimental and Clinical Traumatic Brain Injury. Int. J. Mol. Sci. 22(7) (2021). 10.3390/ijms22073582 10.3390/ijms22073582 PMC8037220 33808272

[9] Barritault D, Gilbert-Sirieix M, Rice KL, Sineriz F, Papy-Garcia D, Baudouin C, Desgranges P, Zakine G, Saffar JL, van Neck J (2017). RGTA(R) or ReGeneraTing Agents mimic heparan sulfate in regenerative medicine: from concept to curing patients. Glycoconj. J..

[10] Lee JY, Xu K, Nguyen H, Guedes VA, Borlongan CV, Acosta SA (2017). Stem Cell-Induced Biobridges as Possible Tools to Aid Neuroreconstruction after CNS Injury. Front. Cell. Dev. Biol..

[11] Rocco ML, Soligo M, Manni L, Aloe L (2018). Nerve Growth Factor: Early Studies and Recent Clinical Trials. Curr. Neuropharmacol..

[12] Bartus, R.T., Johnson, E.M. Jr.: Clinical tests of neurotrophic factors for human neurodegenerative diseases, part 1: Where have we been and what have we learned? Neurobiol. Dis. 97(Pt B), 156–168 (2017). 10.1016/j.nbd.2016.03.027 10.1016/j.nbd.2016.03.027 27063798

[13] Chan SJ, Love C, Spector M, Cool SM, Nurcombe V, Lo EH (2017). Endogenous regeneration: Engineering growth factors for stroke. Neurochem Int..

[14] da Silva Meirelles, L., Simon, D., Regner, A.: Neurotrauma: The Crosstalk between Neurotrophins and Inflammation in the Acutely Injured Brain. Int. J. Mol. Sci. 18(5) (2017). 10.3390/ijms18051082 10.3390/ijms18051082 PMC5454991 28524074

[15] Timaru CM, Stefan C, Iliescu DA, De Simone A, Batras M (2017). Matrix regenerative therapy. Rom J. Ophthalmol..

[16] Dao DT, Anez-Bustillos L, Adam RM, Puder M, Bielenberg DR (2018). Heparin-Binding Epidermal Growth Factor-Like Growth Factor as a Critical Mediator of Tissue Repair and Regeneration. Am. J. Pathol..

[17] Woodbury ME, Ikezu T (2014). Fibroblast growth factor-2 signaling in neurogenesis and neurodegeneration. J. Neuroimmune Pharmacol..

[18] Winkler J, Ramirez GA, Thal LJ, Waite JJ (2000). Nerve growth factor (NGF) augments cortical and hippocampal cholinergic functioning after p75NGF receptor-mediated deafferentation but impairs inhibitory avoidance and induces fear-related behaviors. J. Neurosci..

[19] Abou-El-Hassan H, Sukhon F, Assaf EJ, Bahmad H, Abou-Abbass H, Jourdi H, Kobeissy FH (2017). Degradomics in Neurotrauma: Profiling Traumatic Brain Injury. Methods Mol. Biol..

[20] Hsia HE, Tushaus J, Brummer T, Zheng Y, Scilabra SD, Lichtenthaler SF (2019). Functions of ‘A disintegrin and metalloproteases (ADAMs)’ in the mammalian nervous system. Cell. Mol. Life Sci..

[21] Barritault D, Desgranges P, Meddahi-Pelle A, Denoix JM, Saffar JL (2017). RGTA(R)-based matrix therapy - A new branch of regenerative medicine in locomotion. Joint Bone Spine.

[22] Ikeda Y, Charef S, Ouidja MO, Barbier-Chassefiere V, Sineriz F, Duchesnay A, Narasimprakash H, Martelly I, Kern P, Barritault D, Petit E, Papy-Garcia D (2011). Synthesis and biological activities of a library of glycosaminoglycans mimetic oligosaccharides. Biomaterials.

[23] Khelif Y, Toutain J, Quittet MS, Chantepie S, Laffray X, Valable S, Divoux D, Sineriz F, Pascolo-Rebouillat E, Papy-Garcia D, Barritault D, Touzani O, Bernaudin M (2018). A heparan sulfate-based matrix therapy reduces brain damage and enhances functional recovery following stroke. Theranostics.

[24] Holmes BB, DeVos SL, Kfoury N, Li M, Jacks R, Yanamandra K, Ouidja MO, Brodsky FM, Marasa J, Bagchi DP, Kotzbauer PT, Miller TM, Papy-Garcia D, Diamond MI (2013). Heparan sulfate proteoglycans mediate internalization and propagation of specific proteopathic seeds. Proc. Natl. Acad. Sci. U S A.

[25] Wiley RG, Oeltmann TN, Lappi DA (1991). Immunolesioning: selective destruction of neurons using immunotoxin to rat NGF receptor. Brain Res..

[26] Dashiani, M.G., Kruashvili, L.B., Kh, R., Matatradze, Z., Beselia, S.B.: G.V.: Effects of immunotoxic and electrolytic lesions of medial septal area on spatial short-term memory in rats. Georgian Med News(239), 98–103 (2015) 25802458

[27] Johnson DA, Zambon NJ, Gibbs RB (2002). Selective lesion of cholinergic neurons in the medial septum by 192 IgG-saporin impairs learning in a delayed matching to position T-maze paradigm. Brain Res..

[28] Chang Q, Gold PE (2004). Impaired and spared cholinergic functions in the hippocampus after lesions of the medial septum/vertical limb of the diagonal band with 192 IgG-saporin. Hippocampus.

[29] Gu Z, Yu J, Perez-Polo JR (1998). Long term changes in brain cholinergic markers and nerve growth factor levels after partial immunolesion. Brain Res..

[30] Pongrac JL, Rylett RJ (1998). Molecular mechanisms regulating NGF-mediated enhancement of cholinergic neuronal phenotype: c-fos trans-activation of the choline acetyltransferase gene. J. Mol. Neurosci..

[31] Shin J, Kong C, Lee J, Choi BY, Sim J, Koh CS, Park M, Na YC, Suh SW, Chang WS, Chang JW (2019). Focused ultrasound-induced blood-brain barrier opening improves adult hippocampal neurogenesis and cognitive function in a cholinergic degeneration dementia rat model. Alzheimers Res. Ther..

[32] Fabera P, Parizkova M, Uttl L, Vondrakova K, Kubova H, Tsenov G, Mares P (2019). Adenosine A1 Receptor Agonist 2-chloro-N6-cyclopentyladenosine and Hippocampal Excitability During Brain Development in Rats. Front. Pharmacol..

[33] Gaddum, J.H.: The technique of superfusion. 1953. Br J Pharmacol **120**(4 Suppl), 82-87; discussion 80-81: (1997). 10.1111/j.1476-5381.1997.tb06779.x 10.1111/j.1476-5381.1997.tb06779.x PMC3224274 9142397

[34] Virk MS, Sagi Y, Medrihan L, Leung J, Kaplitt MG, Greengard P (2016). Opposing roles for serotonin in cholinergic neurons of the ventral and dorsal striatum. Proc. Natl. Acad. Sci. U S A.

[35] Adell A, Celada P, Artigas F (2001). The role of 5-HT1B receptors in the regulation of serotonin cell firing and release in the rat brain. J. Neurochem.

[36] Paxinos, G., Watson, C.: The Rat Brain in Stereotaxic Coordinates 7th Edition ed. Elsevier, (2013)

[37] Papy-Garcia D, Barbier-Chassefière V, Rouet V, Kerros M-E, Klochendler C, Tournaire M-C, Barritault D, Caruelle J-P, Petit E (2005). Nondegradative Sulfation of Polysaccharides. Synthesis and Structure Characterization of Biologically Active Heparan Sulfate Mimetics. Macromolecules.

[38] Faivre L, Parietti V, Sineriz F, Chantepie S, Gilbert-Sirieix M, Albanese P, Larghero J, Vanneaux V (2016). In vitro and in vivo evaluation of cord blood hematopoietic stem and progenitor cells amplified with glycosaminoglycan mimetic. Stem Cell. Res. Ther..

[39] Friand V, Haddad O, Papy-Garcia D, Hlawaty H, Vassy R, Hamma-Kourbali Y, Perret G-Y, Courty J, Baleux F, Oudar O, Gattegno L, Sutton A, Charnaux N (2009). Glycosaminoglycan mimetics inhibit SDF-1/CXCL12-mediated migration and invasion of human hepatoma cells. Glycobiology.

[40] Ledoux D, Papy-Garcia D, Escartin Q, Sagot MA, Cao Y, Barritault D, Courtois J, Hornebeck W, Caruelle JP (2000). Human plasmin enzymatic activity is inhibited by chemically modified dextrans. J. Biol. Chem..

[41] Escartin Q, Lallam-Laroye C, Baroukh B, Morvan FO, Caruelle JP, Godeau G, Barritault D, Saffar JL (2003). A new approach to treat tissue destruction in periodontitis with chemically modified dextran polymers. FASEB J..

[42] Lallam-Laroye C, Escartin Q, Zlowodzki AS, Barritault D, Caruelle JP, Baroukh B, Saffar JL, Colombier ML (2006). Periodontitis destructions are restored by synthetic glycosaminoglycan mimetic. J. Biomed. Mater. Res. A.

[43] Morvan FO, Baroukh B, Ledoux D, Caruelle JP, Barritault D, Godeau G, Saffar JL (2004). An engineered biopolymer prevents mucositis induced by 5-fluorouracil in hamsters. Am. J. Pathol..

[44] Tong M, Tuk B, Hekking IM, Vermeij M, Barritault D, van Neck JW (2009). Stimulated neovascularization, inflammation resolution and collagen maturation in healing rat cutaneous wounds by a heparan sulfate glycosaminoglycan mimetic, OTR4120. Wound Repair. Regen.

[45] Koelle GB (1954). The histochemical localization of cholinesterases in the central nervous system of the rat. J. Comp. Neurol..

[46] Hedreen JC, Bacon SJ, Price DL (1985). A modified histochemical technique to visualize acetylcholinesterase-containing axons. J. Histochem. Cytochem..

[47] Birthelmer A, Lazaris A, Riegert C, Marques Pereira P, Koenig J, Jeltsch H, Jackisch R, Cassel JC (2003). Does the release of acetylcholine in septal slices originate from intrinsic cholinergic neurons bearing p75(NTR) receptors? A study using 192 IgG-saporin lesions in rats. Neuroscience.

[48] Birthelmer A, Stemmelin J, Jackisch R, Cassel JC (2003). Presynaptic modulation of acetylcholine, noradrenaline, and serotonin release in the hippocampus of aged rats with various levels of memory impairments. Brain Res. Bull..

[49] Orta-Salazar E, Cuellar-Lemus CA, Diaz-Cintra S, Feria-Velasco AI (2014). Cholinergic markers in the cortex and hippocampus of some animal species and their correlation to Alzheimer’s disease. Neurologia.

[50] Schliebs R, Arendt T (2011). The cholinergic system in aging and neuronal degeneration. Behav. Brain Res..

[51] Traissard N, Herbeaux K, Cosquer B, Jeltsch H, Ferry B, Galani R, Pernon A, Majchrzak M, Cassel JC (2007). Combined damage to entorhinal cortex and cholinergic basal forebrain neurons, two early neurodegenerative features accompanying Alzheimer’s disease: effects on locomotor activity and memory functions in rats. Neuropsychopharmacology.

[52] Hernandes MS, de Magalhaes L, Troncone LR (2007). Glycine stimulates the release of labeled acetylcholine but not dopamine nor glutamate from superfused rat striatal tissue. Brain Res..

[53] Macor JE, Burkhart CA, Heym JH, Ives JL, Lebel LA, Newman ME, Nielsen JA, Ryan K, Schulz DW, Torgersen LK (1990). 3-(1,2,5,6-Tetrahydropyrid-4-yl)pyrrolo[3,2-b]pyrid-5-one: a potent and selective serotonin (5-HT1B) agonist and rotationally restricted phenolic analogue of 5-methoxy-3-(1,2,5,6-tetrahydropyrid-4-yl)indole. J. Med. Chem..

[54] Book AA, Wiley RG, Schweitzer JB (1992). Specificity of 192 IgG-saporin for NGF receptor-positive cholinergic basal forebrain neurons in the rat. Brain Res..

[55] Wallenstein S, Zucker CL, Fleiss JL (1980). Some statistical methods useful in circulation research. Circ. Res..

[56] Huynh, M.B., Ouidja, M.O., Chantepie, S., Carpentier, G., Maiza, A., Zhang, G., Vilares, J., Raisman-Vozari, R., Papy-Garcia, D.: Glycosaminoglycans from Alzheimer’s disease hippocampus have altered capacities to bind and regulate growth factors activities and to bind tau. PLoS One. **14**(1), e0209573 (2019). 10.1371/journal.pone.0209573 10.1371/journal.pone.0209573 PMC6319808 30608949

[57] Bolshakov, A.P., Stepanichev, M.Y., Dobryakova, Y.V., Spivak, Y.S., Markevich, V.A.: Saporin from Saponaria officinalis as a Tool for Experimental Research, Modeling, and Therapy in Neuroscience. Toxins (Basel) 12(9) (2020). 10.3390/toxins12090546 10.3390/toxins12090546 PMC7551693 32854372

[58] Gaddum JH (1953). The technique of superfusion. Br. J. Pharmacol. Chemother..

[59] Ahmadian SS, Rezvanian A, Peterson M, Weintraub S, Bigio EH, Mesulam MM, Geula C (2015). Loss of calbindin-D28K is associated with the full range of tangle pathology within basal forebrain cholinergic neurons in Alzheimer’s disease. Neurobiol. Aging.

[60] Mesulam MM, Lalehzari N, Rahmani F, Ohm D, Shahidehpour R, Kim G, Gefen T, Weintraub S, Bigio E, Geula C (2019). Cortical cholinergic denervation in primary progressive aphasia with Alzheimer pathology. Neurology.

[61] Hampel H, Mesulam MM, Cuello AC, Farlow MR, Giacobini E, Grossberg GT, Khachaturian AS, Vergallo A, Cavedo E, Snyder PJ, Khachaturian ZS (2018). The cholinergic system in the pathophysiology and treatment of Alzheimer’s disease. Brain.

[62] Fahnestock M, Shekari A (2019). ProNGF and Neurodegeneration in Alzheimer’s Disease. Front. Neurosci..

[63] Thinschmidt JS, Frazier CJ, King MA, Meyer EM, Papke RL (2005). Septal innervation regulates the function of alpha7 nicotinic receptors in CA1 hippocampal interneurons. Exp. Neurol..

[64] Heckers S, Ohtake T, Wiley RG, Lappi DA, Geula C, Mesulam MM (1994). Complete and selective cholinergic denervation of rat neocortex and hippocampus but not amygdala by an immunotoxin against the p75 NGF receptor. J. Neurosci..

[65] Waite JJ, Chen AD, Wardlow ML, Thal LJ (1994). Behavioral and biochemical consequences of combined lesions of the medial septum/diagonal band and nucleus basalis in the rat when ibotenic acid, quisqualic acid, and AMPA are used. Exp. Neurol..

[66] Moschonas EH, Leary JB, Memarzadeh K, Bou-Abboud CE, Folweiler KA, Monaco CM, Cheng JP, Kline AE, Bondi CO (2021). Disruption of basal forebrain cholinergic neurons after traumatic brain injury does not compromise environmental enrichment-mediated cognitive benefits. Brain Res..

[67] Rose M, Dudas B, Cornelli U, Hanin I (2003). Protective effect of the heparin-derived oligosaccharide C3, on AF64A-induced cholinergic lesion in rats. Neurobiol. Aging.

[68] Rose M, Dudas B, Cornelli U, Hanin I (2004). Glycosaminoglycan C3 protects against AF64A-induced cholinotoxicity in a dose-dependent and time-dependent manner. Brain Res..

[69] Roher AE, Kuo YM, Potter PE, Emmerling MR, Durham RA, Walker DG, Sue LI, Honer WG, Beach TG (2000). Cortical cholinergic denervation elicits vascular A beta deposition. Ann. N Y Acad. Sci..

[70] Elliott RC, Gall CM (2000). Changes in activating protein 1 (AP-1) composition correspond with the biphasic profile of nerve growth factor mRNA expression in rat hippocampus after hilus lesion-induced seizures. J. Neurosci..

[71] Meddahi A, Benoit J, Ayoub N, Sezeur A, Barritault D (1996). Heparin-like polymers derived from dextran enhance colonic anastomosis resistance to leakage. J. Biomed. Mater. Res..

[72] Meddahi A, Bree F, Papy-Garcia D, Gautron J, Barritault D, Caruelle JP (2002). Pharmacological studies of RGTA(11), a heparan sulfate mimetic polymer, efficient on muscle regeneration. J. Biomed. Mater. Res..

[73] Meddahi A, Lemdjabar H, Caruelle JP, Barritault D, Hornebeck W (1995). Inhibition by dextran derivatives of FGF-2 plasmin-mediated degradation. Biochimie.

[74] Desgranges P, Barbaud C, Caruelle JP, Barritault D, Gautron J (1999). A substituted dextran enhances muscle fiber survival and regeneration in ischemic and denervated rat EDL muscle. FASEB J..

[75] Yamauchi H, Desgranges P, Lecerf L, Papy-Garcia D, Tournaire MC, Moczar M, Loisance D, Barritault D (2000). New agents for the treatment of infarcted myocardium. FASEB J..

[76] Tardieu M, Gamby C, Avramoglou T, Jozefonvicz J, Barritault D (1992). Derivatized dextrans mimic heparin as stabilizers, potentiators, and protectors of acidic or basic FGF. J. Cell. Physiol..

[77] Charef S, Petit E, Barritault D, Courty J, Caruelle JP (2007). Effects on coagulation of a synthetic heparan mimetic given intraperitoneally or orally. J. Biomed. Mater. Res. A.

[78] Morcuende S, Munoz-Hernandez R, Benitez-Temino B, Pastor AM, de la Cruz RR (2013). Neuroprotective effects of NGF, BDNF, NT-3 and GDNF on axotomized extraocular motoneurons in neonatal rats. Neuroscience.

[79] Cui Q (2006). Actions of neurotrophic factors and their signaling pathways in neuronal survival and axonal regeneration. Mol. Neurobiol..

[80] Maeda N (2010). Structural variation of chondroitin sulfate and its roles in the central nervous system. Cent. Nerv. Syst. Agents Med. Chem..

[81] Takeda A, Onodera H, Sugimoto A, Itoyama Y, Kogure K, Rauvala H, Shibahara S (1995). Induction of heparin-binding growth-associated molecule expression in reactive astrocytes following hippocampal neuronal injury. Neuroscience.

[82] Cassel JC, Jeltsch H, Neufang B, Lauth D, Szabo B, Jackisch R (1995). Downregulation of muscarinic- and 5-HT1B-mediated modulation of [3H]acetylcholine release in hippocampal slices of rats with fimbria-fornix lesions and intrahippocampal grafts of septal origin. Brain Res..

[83] Hu XJ, Wang FH, Stenfors C, Ogren SO, Kehr J (2007). Effects of the 5-HT1B receptor antagonist NAS-181 on extracellular levels of acetylcholine, glutamate and GABA in the frontal cortex and ventral hippocampus of awake rats: a microdialysis study. Eur. Neuropsychopharmacol..

[84] Srivastava N, Seth K, Srivastava N, Khanna VK, Agrawal AK (2008). Functional restoration using basic fibroblast growth factor (bFGF) infusion in Kainic acid induced cognitive dysfunction in rat: neurobehavioural and neurochemical studies. Neurochem Res..

[85] Schwartz NB, Domowicz MS (2018). Proteoglycans in brain development and pathogenesis. FEBS Lett..

[86] Maiza A, Chantepie S, Vera C, Fifre A, Huynh MB, Stettler O, Ouidja MO, Papy-Garcia D (2018). The role of heparan sulfates in protein aggregation and their potential impact on neurodegeneration. FEBS Lett..

[87] Sepulveda-Diaz JE, Alavi Naini SM, Huynh MB, Ouidja MO, Yanicostas C, Chantepie S, Villares J, Lamari F, Jospin E, van Kuppevelt TH, Mensah-Nyagan AG, Raisman-Vozari R, Soussi-Yanicostas N, Papy-Garcia D (2015). HS3ST2 expression is critical for the abnormal phosphorylation of tau in Alzheimer’s disease-related tau pathology. Brain.

[88] Parent MB, Baxter MG (2004). Septohippocampal acetylcholine: involved in but not necessary for learning and memory?. Learn. Mem..

[89] Ricceri L (2003). Behavioral patterns under cholinergic control during development: lessons learned from the selective immunotoxin 192 IgG saporin. Neurosci. Biobehav Rev..

[90] Wang, Q., Xiang, B., Deng, W., Wu, J., Li, M., Ma, X., Wang, Y., Jiang, L., McAlonan, G., Chua, S.E., Sham, P.C., Hu, X., Li, T.: Genome-wide association analysis with gray matter volume as a quantitative phenotype in first-episode treatment-naive patients with schizophrenia. PLoS One. **8**(9), e75083 (2013). 10.1371/journal.pone.0075083 10.1371/journal.pone.0075083 PMC3782493 24086445

